# NLRP3 Regulates IL-4 Expression in TOX^+^ CD4^+^ T Cells of Cutaneous T Cell Lymphoma to Potentially Promote Disease Progression

**DOI:** 10.3389/fimmu.2021.668369

**Published:** 2021-06-16

**Authors:** Enrique Huanosta-Murillo, Marcela Alcántara-Hernández, Brenda Hernández-Rico, Georgina Victoria-Acosta, Patricia Miranda-Cruz, María Antonieta Domínguez-Gómez, Fermín Jurado-Santacruz, Genaro Patiño-López, Vadim Pérez-Koldenkova, Alam Palma-Guzmán, Paula Licona-Limón, Ezequiel M. Fuentes-Pananá, Alicia Lemini-López, Laura C. Bonifaz

**Affiliations:** ^1^ Unidad de Investigación Médica en Inmunoquímica, Hospital de Especialidades Centro Médico Nacional Siglo XXI, Instituto Mexicano del Seguro Social, Mexico City, Mexico; ^2^ Departamento de Inmunología, Escuela Nacional de Ciencias Biológicas, Instituto Politécnico Nacional, Mexico City, Mexico; ^3^ Department of Microbiology and Immunology, Stanford University School of Medicine, Stanford, CA, United States; ^4^ Unidad de Investigación, Hospital Juárez de México, Mexico City, Mexico; ^5^ Departamento de Biología Celular, Centro de Investigación y Estudios Avanzados, Instituto Politécnico Nacional, Mexico City, Mexico; ^6^ Centro Dermatológico Dr. Ladislao de la Pascua, Secretaría de Salud de la Ciudad de México, Mexico City, Mexico; ^7^ Laboratorio de Investigación en Inmunología y Proteómica, Sección de Biología Celular de Linfocitos, Unidad de Hemato-Oncología e Investigación Hospital Infantil de México Federico Gómez, Mexico City, Mexico; ^8^ Laboratorio Nacional de Microscopía Avanzada, División de Desarrollo de la Investigación, Centro Médico Nacional Siglo XXI, Instituto Mexicano del Seguro Social, Mexico City, Mexico; ^9^ Laboratorio de Histología, Coordinación de Investigación en Salud, Centro Médico Nacional Siglo XXI, Instituto Mexicano del Seguro Social, Mexico City, Mexico; ^10^ Departamento de Biología Celular y del Desarrollo, Instituto de Fisiología Celular, Universidad Nacional Autónoma de México, Mexico City, Mexico; ^11^ Unidad de Investigación en Virología y Cáncer, Hospital Infantil de México Federico Gómez, Mexico City, Mexico; ^12^ Servicio de Dermatología, Hospital de Especialidades Centro Médico Nacional Siglo XXI, Instituto Mexicano del Seguro Social, Mexico City, Mexico

**Keywords:** Cutaneous T cell lymphoma, Skin lesion, NLRP3, T helper T cell, Th2 cytokines, Interleukin-4

## Abstract

In cutaneous T cell lymphoma (CTCL), a dominant Th2 profile associated with disease progression has been proposed. Moreover, although the production and regulation of IL-4 expression during the early stages of the disease may have important implications in later stages, these processes are poorly understood. Here, we demonstrate the presence of TOX^+^ CD4^+^ T cells that produce IL-4^+^ in early-stage skin lesions of CTCL patients and reveal a complex mechanism by which the NLRP3 receptor promotes a Th2 response by controlling IL-4 production. Unassembled NLRP3 is able to translocate to the nucleus of malignant CD4^+^ T cells, where it binds to the human *il-4* promoter. Accordingly, IL-4 expression is decreased by knocking down and increased by promoting the nuclear localization of NLRP3. We describe a positive feedback loop in which IL-4 inhibits NLRP3 inflammasome assembly, thereby further increasing its production. IL-4 induced a potentially malignant phenotype measured based on TOX expression and proliferation. This mechanism of IL-4 regulation mediated by NLRP3 is amplified in late-stage CTCL associated with disease progression. These results indicate that NLRP3 might be a key regulator of IL-4 expression in TOX^+^ CD4^+^ T cells of CTCL patients and that this mechanism might have important implications in the progression of the disease.

## Introduction

Cutaneous T cell lymphoma (CTCL) represents a group of primary lymphomas in which Mycosis fungoides (MF) is the most common clinical variant (1). CTCL begins as skin patches and plaques (early stage) that may progress into tumors (late stage) (2). Retrospective studies show great variability in the time course of the disease, and early-stage lesions can persist for months or years without progression (2, 3). The slow evolution and chronic nature of skin lesions suggest that there is a strong interplay between malignant cells and the immune system.

In the early stages of CTCL, malignant T cells are mixed with tumor-infiltrating lymphocytes (TILs) (4, 5). Recent studies reported that the nuclear factor TOX (thymocyte selection-associated high mobility group box) is overexpressed in malignant T cells from CTCL lesions (6, 7). Therefore, TOX expression can be used to discriminate between malignant T cells and TILs. In addition, clonal expression of certain TCRVβ chains was reported to be associated with malignant clones (8). However, the association between clonality and TOX expression has not been explored.

In many types of neoplasms, Th1 responses are necessary for effective antitumor defense (9, 10). Meanwhile, Th2 responses have a detrimental role in inhibiting polarization towards a Th1 profile, and a shift from Th1 to Th2 mediated by the dominance of Th2 cytokines has been proposed to explain the disease progression (2). However, in CTCL, TOX^+^ Th2 cells are present beginning in the early stages of the disease and fade the boundary between a Th1 and Th2 shift (11). Therefore, enhanced expression of Th2 cytokines and the mechanisms regulating their expression in CD4^+^ T cells may be more illustrative of the turning point between CTCL control and progression. Indeed, IL-4 and IL-13 Th2 cytokines have protumoral activity in several types of tumors, including CTCL (12–15).

GATA-3 is the master transcription factor (TF) that regulates the expression of the Th2 locus in mice and humans (16). A novel mechanism for the regulation of IL-4 expression was also described in mouse Th2 cells, in which the unassembled form of NLRP3 is translocated to the nucleus by importin karyopherin α2 and then binds to IRF4 and acts as a TF to activate the *il-4* promoter, thus favoring Th2 polarization (17). NLRP3-mediated production of IL-4 was also described in human macrophages (18), suggesting that this mechanism is conserved between mice and humans. Furthermore, IL-4 inhibits NLRP3 inflammasome assembly in monocytes and the production of inflammatory cytokines (19). However, few studies have explored this mechanism in human T cells, and it is not clear whether it contributes to diseases associated with these cells.

NLRP3 expression depends on the signal transducer STAT5, and STAT5 is constitutively active in CTCL lesions beginning in the early stages of the disease (20). Taken together, these results indicate that NLRP3 could be expressed in the CD4^+^ T cells present in the skin lesions of CTCL patients and hence could be a regulator of Th polarization. In this study, we documented the expression of IL-4 in TOX^+^ CD4^+^ T cells present in the skin lesions of CTCL patients. Although we could not find evidence of GATA3 driving the expression of IL-4, nuclear NLRP3 consistently marked specimens with high TOX and IL-4 expression. Furthermore, we demonstrated in vitro that IL-4 creates a positive feedback loop that inhibits cytoplasmic activation of the NLRP3 signalosome, thereby promoting NLRP3 nuclear translocation and regulating *il-4* expression. Finally, the density of IL-4^+^ TOX^+^ CD4^+^ T cells and the levels of expression of these molecules significantly marked lesion progression from plaque to tumor.

## Materials and Methods

### CTCL Patients and Controls

Patients were recruited from the Centro Dermatológico “Dr. Ladislao de la Pascua”, the pathology departments of Instituto Nacional de Ciencias Médicas y Nutrición “Salvador Zubirán” and Hospital de Especialidades of Centro Médico Nacional Siglo XXI. The study was conducted according to the principles detailed in the Declaration of Helsinki and was approved by the Ethics and Scientific Committees of the participating hospitals. Sixty-one patients (53 in the plaque stage and 8 in the tumor stage) were included in the study. All patients fulfilled the diagnostic criteria for CTCL according to the TNM-ISCL (the tumor-node-metastasis scale, proposed by the International Society of Cutaneous Lymphomas) (21). We also collected control skin (CS) samples (n=12) from remnant skin derived from plastic surgeries that were free of dermatologic pathologies and lesions from patients with psoriasis (n=10) as a comparative control of a benign inflammatory disease (BID) and atopic dermatitis (n=12) as an example of a Th2 BID (**Supplementary Figure 1**). All participants signed informed consent forms. Patient information is listed in **Supplementary Tables 1, 2**.

### Preparation of Skin Cells

CTCL, CS and BID tissue samples were either fixed and embedded in paraffin (see below) or cultured to obtain cell suspensions. To obtain cellular suspensions, the biopsies were washed in phosphate-buffered saline and the adipose tissue was removed. Samples were then fragmented into pieces of equal size and treated overnight with Dispase II (1 mg/mL; Roche, Mannheim, Germany). The dermis and epidermis were separated and cultured in RPMI 1640 (Life Technologies, CA, USA) using equal volumes of medium and then incubated for 120 h. CD4^+^ T cells were isolated by positive selection (Miltenyi Biotec, Gladbach, Germany), and approximately 50000 cells were obtained on average from one 6 mm biopsy of a CTCL patient and from 15-20 mm biopsies of CS. Cells from skin cultures were labeled with CellTrace Violet (Thermo Scientific, MA, USA) and cultured for an additional 3 days. Cells from skin cultures were either directly immunophenotyped or activated for intracellular cytokine detection.

### Cell Lines

HTB-176 (H9) human CTCL cell line derivatives of the HuT 78 (ATCC Cat# HTB-176, RRID:CVCL_1240) and Jurkat T cell line (CLS Cat# 300223/p849_Jurkat_E61, RRID:CVCL_0367) were obtained from ATCC, USA. HTB-176 cells were cultured in supplemented RPMI 1640 High Glucose medium (ATCC, USA), and Jurkat cells were cultured in supplemented RPMI 1640 medium (ATCC, USA). Cells were grown at 37 °C and 5% CO_2_ in a humidified incubator.

### Preparation of Tissue Sections and Cells

Skin biopsies were embedded in paraffin. Five μm sections of tissue were cut into charged glass slides (Superfrost Plus Yellow, Sigma Aldrich, MO, USA) and rehydrated. Heat-induced antigen retrieval was performed using citrate buffer at a pH of 6.0 (sodium citrate 10 μM) at 90°C for 20 min. HTB-176 cells were placed directly onto charged glass slides.

### Immunofluorescence of Tissue Sections and Cells

The sections were permeabilized (bovine serum albumin, 10 mg/mL; horse serum, 5%; sodium azide, 0.02%; Triton, 0.5%) for 2 h and incubated with the following primary antibodies overnight at room temperature (RT): anti-IL-4 (Santa Cruz Biotechnology Cat# sc-1260, RRID:AB_2128970), anti-IL-13 (Abcam Cat# ab106732, RRID:AB_10867235), anti-TOX (Thermo Fisher Scientific Cat# PA5-30328, RRID:AB_2547802), anti-TCRVβ22 (Beckman Coulter Cat# IM1484, RRID:AB_131022), anti-IFN**γ** (BioLegend Cat# 507501, RRID:AB_2122340), anti-NRLP3 (R&D systems, Cat# MAB7578, RRID: AB_2889405), anti-karyopherin α2 (BD Biosciences Cat# 610485, RRID:AB_397855), and anti-GATA-3 (Abcam Cat# ab106625, RRID:AB_10887935). Subsequently, the slides were incubated for 2 h at RT with the following fluorescent secondary antibodies: anti- rat IgG(H+L) AF488(Thermo Fisher Scientific Cat# A-11006, RRID:AB_2534074), anti-rat IgG(H+L) 594 (Jackson ImmunoResearch Labs Cat# 712-585-153, RRID:AB_2340689), Biotin-SP anti-Goat IgG (H+L) (Jackson ImmunoResearch Labs Cat# 705-065-147, RRID:AB_2340397), anti-rabbit IgG (H+L)(Jackson ImmunoResearch Labs Cat# 711-585-152, RRID:AB_2340621), anti-rabbit IgG (H+L)(Jackson ImmunoResearch Labs Cat# 711-545-152, RRID:AB_2313584) and anti-mouse (H+L) (Jackson ImmunoResearch Labs Cat# 715-605-151, RRID:AB_2340863). The antibodies used are listed in **Supplementary Table S3**. The nuclei were counterstained with Hoechst (Invitrogen, CA, USA) for 10 min. The sections were mounted with Vectashield (Vector Laboratories, CA, USA), and images were acquired on a Nikon Ti Eclipse inverted confocal microscope (Nikon, ME, NY) using NIS Elements v.4.50 as well as a Leica TCS SP8x with WLL and HyD detectors (Leica, Wetzlar, Germany), and analyzed using ImageJ Software (ImageJ software, National Institutes of Health). One scale bar is included in every set of images unless we present a magnification of the microphotograph. Three 40x microphotographs were taken for each patient and control, and the expression density was measured based on the positive area using the integrated density parameter, for a total area of 300 μm. On the x-axis of the graphs, values report the logarithm of the average intensity × area of each stained region.

Three-dimensional (3D) cell models were acquired on a Nikon Ti Eclipse inverted confocal microscope (Nikon, ME, NY) using NIS Elements v.4.50, and 20× micrographs with 4× magnification were sectioned with an average of 15 slices. The reconstructions were created with the ImageJ Software program.

### Immunohistochemistry

The tissues were incubated in 4% hydrogen peroxide for 5 min and then with the following primary antibodies for 30 min: anti-TOX (Thermo Fisher Scientific Cat# PA5-30328, RRID:AB_2547802), anti-TCRVβ22 (Beckman Coulter Cat# IM1484, RRID:AB_131022), anti-NRLP3 (R&D Systems, Cat# MAB7578, RRID: AB_2889405). Afterwards, the slides were incubated for 40 min at RT with the following secondary antibodies: anti-mouse and anti-rabbit micro polymer alkaline phosphatase-linked IG antibody (Bio SB Cat# BSB-0351, RRID: AB_2891237) or anti-rabbit horseradish peroxidase-linked IgG antibody (Leica, Cat# DS9800, RRID: AB_2891238). Finally, substrate-AP chromogen (Bio SB) or diaminobenzidine hydrate (Leica) was applied for 1 min. The tissues were counterstained with Gill’s hematoxylin for 1 min and dehydrated with 96% ethanol and xylene. Tissues were preserved by applying a coverslip and cloth (Merck, Darmstadt, Germany). Images were acquired with an Aperio CS2 Scanner (Leica) at 20x and analyzed with the software Aperio ImageScope (RRID: SCR:014311).

### Chromatin Immunoprecipitation Assay

ChIP assays were performed with 3 × 10^6^ HTB-176 cells. First, the cells were cross-linked with 1% formaldehyde and then quenched by adding 125 mM glycine at RT. The cells were then lysed, and chromatin was sheared into 200- to 500-bp fragments using a sonicator (SONICS Vibra-Cell, CO, USA). ChIP was performed using the following antibodies: 4 μg anti-NRLP3 (R&D Systems, MI, USA), 4 μg anti-IRF4 (BioLegend Cat# 646411, RRID:AB_2728477), 4 μg of anti-IgG (Thermo Fisher Scientific Cat# 13-4724-85, RRID:AB_470090) or 4 μg anti-CTCF (Millipore Cat# 07-729, RRID:AB_441965). Immunoprecipitated DNA was analyzed by PCR using the primers listed in **Supplementary Table S4**. An anti-CTCF antibody and the promoter region of the IGF2 gene that harbors a CTCF binding site were used as positive controls. This antibody was also used as a negative control for the NRLP3-specific interaction with the IL-4 promoter.

### siRNA Knockdown of NLRP3

siRNA knockdown of NLRP3 was performed in the HTB-176 cell line. HTB-176 cells were transfected with 100 nmol of short interfering RNA against NLRP3 (siNLRP3, Qiagen, Germany) or scrambled control RNA (siScr) (Integrated DNA Technologies, IO, USA) in the presence of HiPerfect reagent (Qiagen, Germany) or Lipofectamine (Thermo Scientific, MA, USA). NLRP3 knockdown was confirmed by Western blot and immunofluorescence 72 h after transfection.

### MCC950 and IL-4 Treatment

NLRP3 inflammasome assembly inhibition was performed in HTB-176 cells treated with MCC950 (5 μM, Avistron chemistry, UK) as previously described (22). HTB-176 cells and skin biopsy cells were treated with IL-4 (20 ng/mL, Peprotech, NJ, USA) for 3 h plus brefeldin A and monensin for 6 h. In both cases, inflammasome assembly inhibition and IL-4 stimulation cells were activated with simultaneous additions of PMA/ionomycin (Cell Stimulation Cocktail, 500x, eBioscience, CA, USA).

In addition, skin cell suspensions were stimulated or not with IL-4 (20 ng/mL) and labeled with CellTrace Violet (CTV) for 3 days. After that, the cells were recovered and stained for flow cytometry to evaluate their proliferation by dilution of CTV or Ki67 expression (anti-Ki67, BioLegend Cat# 350522, RRID:AB_2563863) and their cytokine and transcription factor expression.

### Flow Cytometry

Skin cell suspensions and HTB-176 cells were stained for flow cytometry as previously described (23). The following antibodies were used: anti-CD3 (BioLegend Cat# 317307, RRID:AB_571912), anti-CD4 (BD Biosciences Cat# 560158, RRID:AB_1645478), anti-IL-4 (BioLegend Cat# 500822, RRID:AB_961404), anti-T-bet (BioLegend Cat# 644805, RRID:AB_1595593), anti-GATA-3 (BD Biosciences Cat# 560163, RRID:AB_1645302), anti-IFN**γ** (BD Biosciences Cat# 557643, RRID: AB_396760) and anti-TOX (Thermo Fisher Scientific Cat# 50-6502-82, RRID:AB_2574265). A full list of the antibodies used is included in **Supplementary Table S3**. Samples were acquired using a FACS Canto II system (BD Bioscience, NJ, USA).

### Cytokine Quantification by Cytometric Bead-Based Assay

For cytokine quantification, supernatants were recovered after 72 h of culture. Cytokines present in the skin culture supernatants were detected using a Th1, Th2, Th17 and Th22 FlowCytomix Multiplex (eBioscience, USA). Samples were washed and acquired using a FACSCalibur system (BD Bioscience, USA).

### Enrichment of Nuclear and Cytoplasmic Fractions

A total of 4×10^6^ cells were harvested and washed with 1 mL PBS pH 7.4 at 300 g for 3 min at 4 °C 3 times. We added 75 µL of NP-40 buffer (50 mM Tris-HCl pH 7.5, 100 mM NaCl, 2 mM EDTA, 0.6% NP-40) and protease inhibitors, and then the cells were vortexed gently for 5 s and incubated for 5 min at 4°C with shaking. Then, the cells were centrifuged at 400 g for 5 min at 4°C. Supernatants were collected and labeled as cytoplasmic fractions. We added NP-40 buffer again, repeated the previous steps, and joined the two supernatants corresponding to the cytoplasmic fraction. The pellets were resuspended in 125 µL of buffer 2 (0.35 M sucrose, 0.5 mM MgCl2) and protease inhibitors and incubated for 5 min at 4°C. After incubation, they were centrifuged at 8×10^3^ rpm for 5 min at 4°C to eliminate debris, and the supernatant was collected as a nuclear fraction. Both the cytoplasmic and nuclear fractions were stored at -20°C.

### Western Blot Analysis

Cell lysates were quantified by the Bradford method and then denatured in loading buffer by heating for 5 min at 99°C. Fifty micrograms of protein was loaded and separated by 12% SDS-PAGE and transferred to NC membranes. The membrane was blocked for 2 h with Intercept (PBS) Blocking Buffer and Intercept T20 (PBS) Antibody Diluent (LI-COR Biotechnology, USA), incubated overnight with anti-NLRP3 (R&D Systems, MAB7578, RRID: AB_2889405) and anti-TOX (Thermo Fisher Scientific Cat# PA5-30328, RRID:AB_2547802) antibodies and incubated for 2 h with anti-Actin (GeneTex Cat# GTX109639, RRID:AB_1949572) antibodies. The membrane was washed with PBS-Tween 0.1% for 10 min 3 times. After washing, the membrane was incubated with anti-rat (LI-COR Biosciences Cat# 926-32219, RRID:AB_1850025) and anti-rabbit (LI-COR Biosciences Cat# 926-32211, RRID:AB_621843) secondary antibodies for 2 h, washed 3 times with PBS-Tween 0.1% and imaged with an Odyssey Infrared Imaging System (LI-COR Biotechnology, USA).

For NLRP3 Western blotting, immunoprecipitation of the cell extracts with protein G was performed with anti-NLRP3 (R&D Systems, MAB7578, RRID: AB_2889405). Then, the precipitates were denatured in loading buffer, and the aforementioned steps were continued.

### Neutralization of IL-4

HTB-176 cells were activated with phytohemagglutinin and treated with anti-IL-4 (10 μg/mL, R&D Systems, MAB304, RRID: AB_2889404) for 3 days in culture plus brefeldin A and monensin for 6 h. Cells were stained for immunofluorescence evaluation of TOX (Thermo Fisher Scientific Cat# PA5-30328, RRID:AB_2547802) and Ki67 (Thermo Fisher Scientific Cat# 14-5698-82, RRID:AB_10854564).

### Statistical Analysis

Central tendency measures were used to describe the study. The data are expressed as the mean ± SEM, and unless otherwise indicated, the data were normalized using a logarithmic 10 transformation. To assess statistically significant differences in the expression of markers, an unpaired Student’s t-test was used for two groups, and a one-way ANOVA with Bonferroni’s multiple comparison test was used for more than two groups. Finally, Spearman’s test was used to analyze the correlations between two data sets. All p values equal to or below 0.05 were considered statistically significant. Analyses were performed using GraphPad Prism 7.0 (San Diego, CA, USA).

## Results

### IL-4 Is Produced by TOX^+^ CD4^+^ T Cells in the Skin Lesions of Early-Stage CTCL Patients

**Figure 1:**
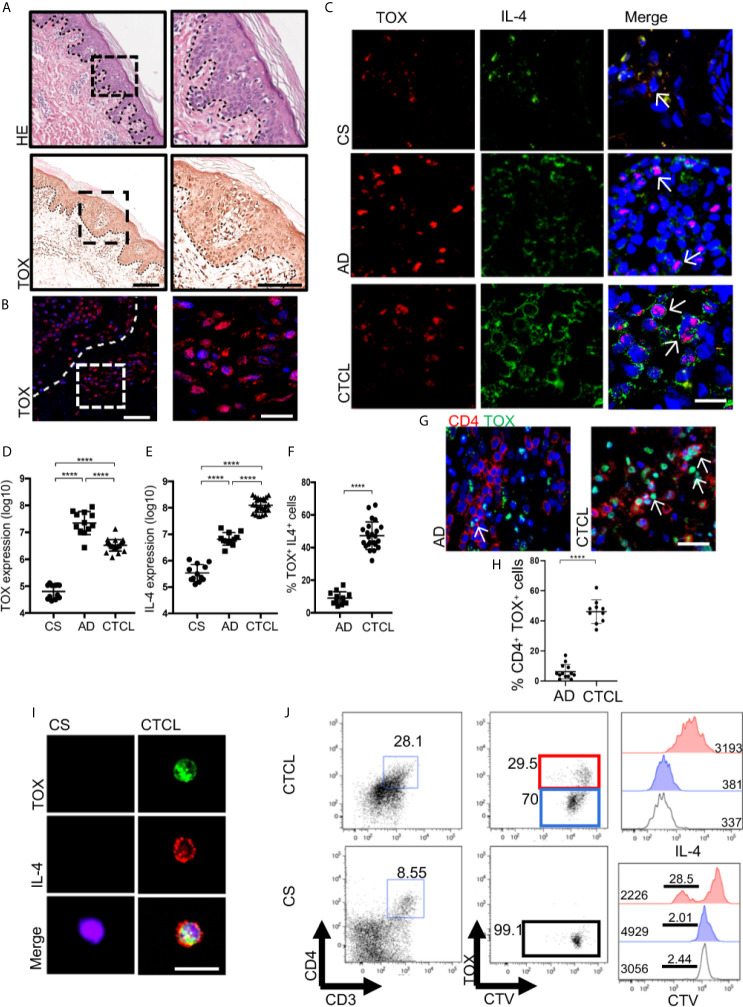
IL-4 is produced by TOX^+^ CD4^+^ T cells in the skin lesions of early-stage CTCL patients. **(A)** Hematoxylin-eosin (HE) staining and immunohistochemistry (IHC) of TOX in CTCL plaques. **(B)** Representative immunofluorescence (IF) of TOX (AF-594) in the Pautrier microabscesses area of CTCL plaques. **(C)** Representative IF of TOX (AF-594) and IL-4 (AF-488) in CTCL plaques, AD and CS. Quantification of the total expression of **(D)** TOX, **(E)** IL-4 and **(F)** percentage of TOX^+^ IL-4^+^ cells in the microphotographs of CTCL plaques (n = 23), AD (n = 12) and CS (n = 12). **(G)** Representative IF of TOX (AF-488) and CD4 (AF-594) in CTCL plaques and AD. Quantification of the percentage of **(H)** CD4^+^ TOX^+^ cells in the microphotographs of CTCL plaques (n = 23) and AD (n = 2). **(I)** Representative IF of TOX (AF-488) and IL-4 (AF-594) in CD4^+^ cells isolated from CTCL plaques and CS (n = 5). **(J)** Representative plot of proliferating CellTrace Violet (CTV) TOX^+^ cells and histogram of IL-4 and CTV from TOX^+^ and TOX^-^ cells gated on CD4^+^ cells (n = 4). Bar graph of IL-4 MFI from TOX^-^ cells (blue, CS) and TOX^+^ cells (red, CTCL). The dotted line in A and B indicates the dermoepidermal junction. Arrows indicate coexpression of markers in the different staining. Error bars = SEM. ****P < 0.0001. Scale bars= 20 nm.

Because a switch from a Th1 to Th2 immune response could explain the progression of CTCL lesions, we assessed the expression of Th1 and Th2 cytokines and master regulatory TFs in the skin lesions of early-stage CTCL patients. Although we found that early lesions were characterized by a mixed Th1/Th2 profile, we observed the presence of large IL-4^+^ CD4^+^ T cells in the epidermis that were not present in the control samples (**Supplementary Figures 2, 3**). To determine whether these cells corresponded to potentially malignant T cells, we evaluated the expression of the nuclear factor TOX in the Pautrier microabscesses and in the basement membrane of CTCL plaques (**Figures 1A, B**). As shown in **Figures 1C–E**, TOX and IL-4 expression was significantly higher in the epidermis of CTCL plaques, whereas it was nearly absent in CS or psoriasis (**Supplementary Figure 4**). Surprisingly, we observed higher TOX expression but significantly lower IL-4 expression in Atopic Dermatitis (AD) as a control of a Th2 BID (**Figures 1C–E**). Additionally, the percentage of TOX^+^ IL-4^+^ positive cells was significantly higher in CTCL than in AD (**Figure 1F**), and the expression of TOX was not restricted to CD4^+^ T cells in AD (**Figures 1G, H**), which is contrary to that in CTCL. Importantly, we observed the expression of IL-4 in TOX^+^ cells even in early-stage CTCL lesions. In contrast, IFNγ was detected in TOX^-^ cells and showed higher expression in CTCL plaques than in CS (**Supplementary Figures 5A–C**). Furthermore, we observed an increase in IL-13 expression in CTCL lesions compared with CS (**Supplementary Figures 5D, E**). We observed clear nuclear TOX expression and abundant cytoplasmic expression of IL-4 in CD4^+^ T cells isolated from CTCL plaques, while this pattern was not detected in CD4^+^ T cells isolated from CS (**Figure 1I**). We observed the presence of proliferating IL-4^+^ CD4^+^ CD3^+^ T cells in cell cultures of CTCL plaques but not in CS, which were also TOX^+^ (**Figure 1J**). Next, to determine the association of TOX expression and T cell clonality in early-stage CTCL, we evaluated the expression of TCRVβ22 and observed variation ranging from 14% to 75%. In patients with high clonal expression of TCRVβ22, positive cells were observed in the Pautrier microabscesses of CTCL plaques (**Supplementary Figure 6A**). Interestingly, we observed a strong positive correlation between the expression of TOX and TCRVβ22, with the number of double-positive cells increasing in patients showing greater evidence of TCRVβ22 clonal expansions (**Supplementary Figure 6B**). We also observed IL-4 expression in TOX^+^ cells in patients with high TCRVβ22 expression (**Supplementary Figure 6C**). These results indicate that IL-4 is produced by TOX^+^ CD4^+^ T cells in the skin lesions of CTCL patients and that the clonal expansion of these cells provides a Th2 cytokine milieu at early stages of the disease.

### NLRP3 and Karyopherin α2 Display Nuclear and Perinuclear Localization in CTCL Plaques

**Figure 2:**
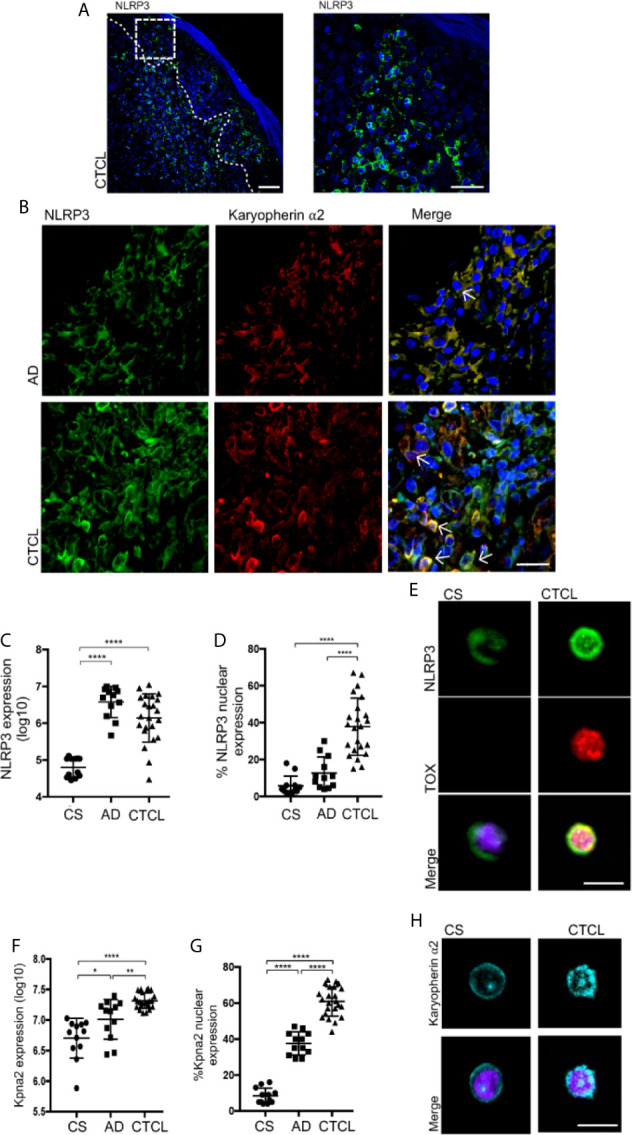
NLRP3 and karyopherin α2 display nuclear and perinuclear localization in CTCL plaques. **(A)** Representative microphotograph showing NLRP3 expression in the Pautrier microabscesses area. **(B)** Representative immunofluorescence (IF) and z-stack reconstructions of NLRP3 (AF-488) and karyopherin α2 (AF-594) expression in CTCL plaques and AD (**Supplementary Videos 1 **and** 2**, respectively). Quantification of the total expression of **(C)** NLRP3 and **(D)** percentage of cells with NLRP3 nuclear expression in the microphotographs of CTCL plaques (n = 23), AD (n = 12), and CS (n = 12). **(E)** Representative IF of NLRP3 (AF-488) and TOX (AF-594) in CD4^+^ T cells isolated from CTCL plaques and CS (n = 5). Quantification of the **(F)** total expression of karyopherin α2 and **(G)** percentage of karyopherin α2^+^ cells in the microphotographs of CTCL plaques (n = 23), AD (n = 12), and CS (n = 12). **(H)** Representative IF of karyopherin α2 in CD4^+^ T cells isolated from CTCL plaques and CS (n = 5). Arrows indicate coexpression of markers in the different staining. Error bars = SEM. *P < 0.05, **P < 0.01, ****P < 0.0001. Scale bars= 20 nm.

Although we observed IL-4 production by TOX^+^ CD4^+^ T cells from CTCL plaques, GATA-3 did not show differences in expression level between the CTCL and CS specimens and showed significantly lower expression in CTCL relative to AD (**Supplementary Figure 2, 3 **and** 6D**), suggesting that GATA-3 was not totally responsible for the basal IL-4 expression observed in CTCL CD4^+^ T cells and indicating the occurrence of alternative regulation mechanisms for IL-4 expression. Since NLRP3 also transcriptionally drives IL-4 expression in mouse T cells and human macrophages (17, 18), we evaluated its expression and localization in the Pautrier microabscesses area and basement membrane of CTCL plaques (**Figure 2A**). We observed significantly higher expression of NLRP3 in early CTCL than in CS and psoriasis as a BID (**Supplementary Figure 6E**). Of note, although AD was also marked by high NLRP3 expression, the signal was not nuclear, whereas with CTCL, a higher percentage of cells presented NLRP3 nuclear localization (**Figures 2B–D** and **Supplementary Videos 1, 2**).

High nuclear expression of NLRP3 in TOX^+^ cells was also observed in CD4^+^ T cells isolated from skin cell cultures of CTCL plaques, while CD4^+^ T cells from CS showed that NLRP3 was mainly localized in the cytosol (**Figure 2E**). We also observed significantly higher expression of karyopherin α2 in CTCL plaques than in CS and AD (**Figure 2F**). Importantly, karyopherin α2 primarily presented cytosolic expression in AD but perinuclear and nuclear expression in CTCL plaques (**Figures 2B, G** and **Supplementary Videos 1, 2**). Nuclear and perinuclear expression of karyopherin α2 was also observed in CD4^+^ T cells isolated from CTCL lesions (**Figure 2H**). These results indicate that NLRP3 and karyopherin α2 display nuclear and perinuclear localization, respectively, suggesting that NLRP3 could be translocated to the nucleus and implicated in the upregulation of IL-4 in CTCL lesions.

### NLRP3 binds to the *il-4* promoter and regulates IL-4 expression in malignant CD4^+^ T cells from CTCL patients.

**Figure 3:**
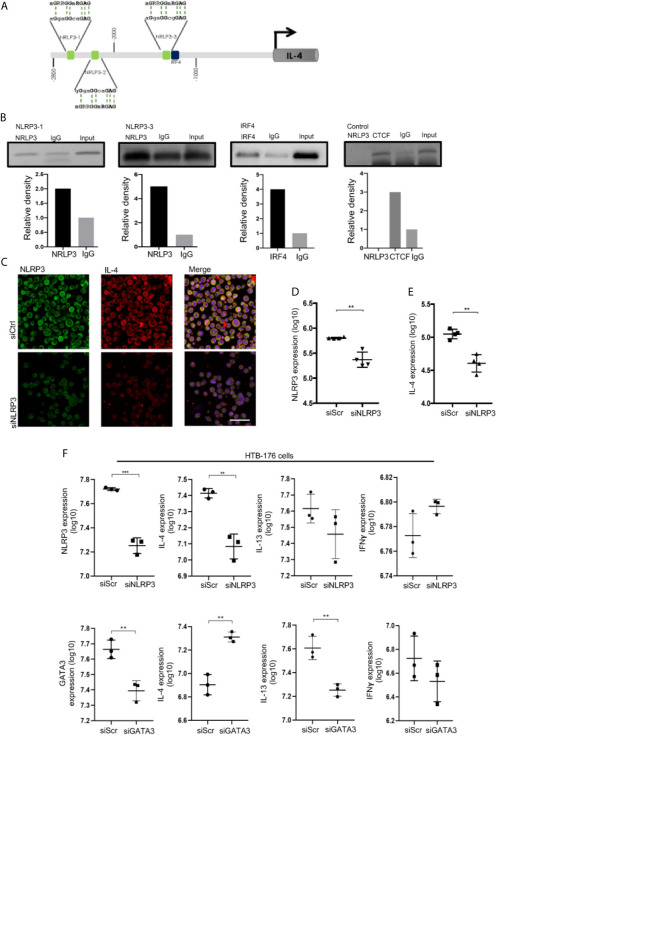
NLRP3 binds to the *il-4* promoter and regulates IL-4 expression in malignant CD4^+^ T cells from CTCL patients. **(A)** Schematic representation of the localization of three putative NRLP3 and one IRF4 binding site in the region from -2800 to -1000 bp relative to the transcription start site in the *il-4* promoter. **(B)** ChIP of NLRP3 and IRF4 in HTB-176 cells, quantification of binding to NRLP3 site 1 (NLRP3-1) and site 3 (NLRP3-3), and binding to IRF4 (n = 2). **(C)** HTB-176 cell line was transfected with siNLRP3 and siScr using HiPerfect, and representative microphotographs depicting NLRP3 (AF-488) and IL-4 (AF-594) expression in transfected cells. Quantification of total expression of **(D)** NLRP3 and **(E)** IL-4 in the microphotographs after transfection (n = 4). **(F)** HTB-176 cell line was transfected with siNLRP3, siGATA3 and siScr using Lipofectamine. Quantification of the total expression of NLRP3, GATA3, IL-4, IL-13 and IFNγ in microphotographs after transfection (n = 3). Error bars = SEM; **P < 0.01; ***P < 0.001. Scale bars= 20 nm.

To evaluate whether NLRP3 may regulate IL-4 expression in malignant CD4^+^ T cells, we used the HTB-176 cell line isolated from the peripheral blood of a patient with Sézary syndrome (24, 25). We first demonstrated that HTB-176 cells also exhibit a NLRP3^+^ karyopherin α2^+^ TOX^+^ CD4^+^ T cell phenotype and that they express IL-4 and IL-13 cytokines at basal levels and upon PMA stimulation (**Supplementary Figures 7A–E**). Although NLRP3 binds to the mouse *il-4* promoter, this mechanism has not been explored in human T cells. To elucidate whether NLRP3 can function as a potential TF in human T cells, we used the consensus motif 5’-nGRRGGnRGAG-3’ published by Bruchard (17) to search for NLRP3-targeted sequences in the *il-4* promoter region between -1 and -3000 bp relative to the transcription start site (TSS), and we identified three putative binding sites (**Figure 3A**). We performed ChIP assays to experimentally test whether these sites were bound by NLRP3, and we detected a weak association with binding site 1 and a strong association with binding site 3, which is closer to the *il-4* TSS. Importantly, we also identified an IRF4 active binding site near binding site 3 of NLRP3 (**Figures 3A, B**).

To directly address the dependency of IL-4 expression on NLRP3 in these cells, we performed a loss-of-function experiment in which we knocked down NLRP3 by transfecting short interfering RNAs (siNLRP3) or scrambled RNA (siScr) as a control. We observed a significant reduction in NLRP3 expression in transfected HTB-176 cells (**Figures 3C, D, F**, **Supplementary Figure 8F**). Notably, a significant reduction in the expression of IL-4 was observed (**Figures 3C, E, F**), although significant changes were not observed in IL-13 or IFNγ expression (**Figure 3F**). Similar results were observed in Jurkat cells, which is another CD4^+^ T cell line (**Supplementary Figure 8A**). Interestingly, the knockdown of GATA-3 increased IL-4 expression but decreased IL-13 expression considerably (**Figure 3F**). As expected, IFNγ expression was not affected (**Figure 3F**). These results were also supported by the phytohemagglutinin (PHA)-dependent overexpression of NLRP3, which resulted in a significant increase in IL-4 expression (**Supplementary Figure 8B**). These data indicate that NLRP3 binds to *il-4* promoter sequences and controls IL-4 levels in malignant CD4^+^ T cells from early CTCL lesions.

## Nuclear Localization of NLRP3 Positively Regulates IL-4 in Malignant CD4^+^ T Cells From CTCL Patients

**Figure 4:**
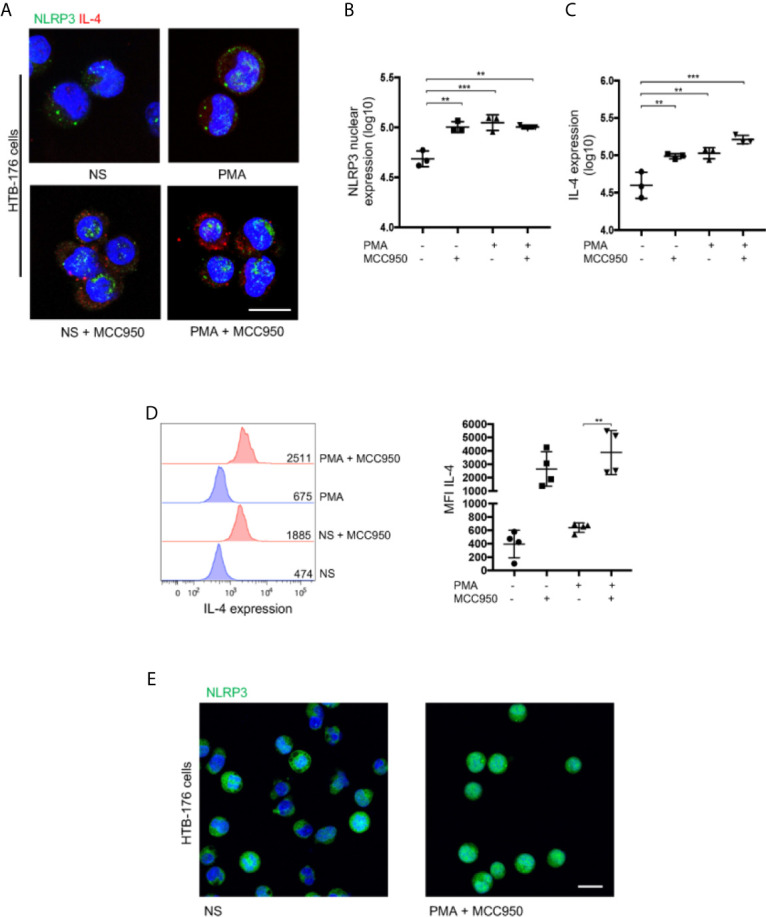
Nuclear localization of NLRP3 positively regulates IL-4 in malignant CD4^+^ T cells from CTCL patients. **(A)** HTB-176 cells were stimulated with PMA/ionomycin with or without an NLRP3 assembly inhibitor (MCC950). Representative immunofluorescence (IF) for NLRP3 (AF-488) and IL-4 (AF-594) expression. Quantification of **(B)** NLRP3 nuclear expression and **(C)** total expression of IL-4 in the microphotographs of the different treatments (n = 4). **(D)** Flow cytometric analysis of cells treated as in **(A)**, representative histogram and MFI plot of IL-4 expression (n = 3). **(E)** Z-stack imaging and reconstruction depicting NLRP3 expression in HTB-176 cells not stimulated or after treatment with PMA plus the NLRP3 assembly inhibitor (**Supplementary Videos 3 **and** 4**, respectively). Error bars = SEM; **P < 0.01; ***P < 0.001. Scale bars= 20 nm.

Nuclear localization of NLRP3 was shown to be necessary for the regulation of IL-4 in mouse Th2 cells rather than cytosolic availability for inflammasome assembly (17). To evaluate whether this is the case in CTCL, we treated HTB-176 cells with the small molecule MCC950, an inhibitor of NLRP3 inflammasome assembly (22). We observed a significant increase in the nuclear localization of NLRP3 in cells treated with MCC950 (**Figures 4A, B**). Remarkably, increased expression of IL-4 was also observed in both unstimulated and PMA/ionomycin-stimulated CD4^+^ T cells after treatment with MCC950 (**Figures 4A, C**). The increase in IL-4 expression was confirmed by flow cytometry, where a significant increase in the mean fluorescence intensity was observed in MCC950-treated cells (**Figure 4D**). The nuclear localization of NLRP3, which increases in the presence of the MCC950 inhibitor, was confirmed by confocal microscopy by Z-stack and reconstruction videos (**Figure 4E** and **Supplementary Videos 3, 4**) and by cytosol and nuclear fractionation of the HTB-176 cell line extracts, in which a decrease in the cytosol fraction but an increase in the nuclear fraction was observed in the presence of the MCC950 inhibitor plus PMA/ionomycin (**Supplementary Figure 9**). Importantly, in the cells treated with MCC950, there was an important increase in the binding of NLRP3 to site 1 of the *il-4* promoter (**Supplementary Figure 10**). These results indicate that inhibiting cytoplasmic inflammasome assembly favors NLRP3 nuclear localization, binding to the *il-4* promoter and positive regulation of IL-4 expression in CD4^+^ T cells from CTCL.

### IL-4 Promotes NLRP3 Nuclear Localization in Malignant CD4^+^ T Cells, and Tumor Lesions Show Augmented NLRP3 Expression in CTCL

**Figure 5:**
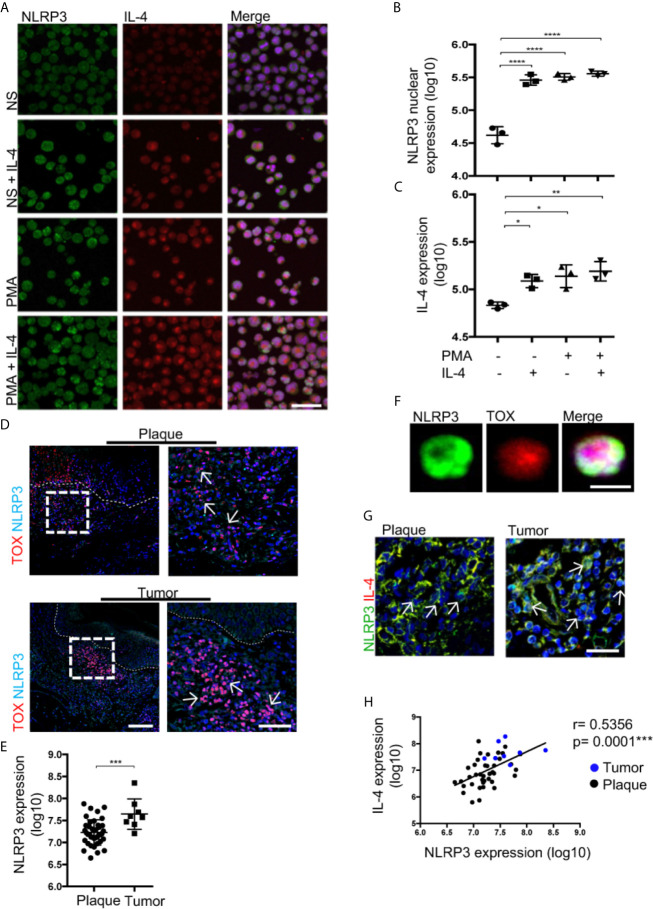
IL-4 promotes NLRP3 nuclear localization in malignant CD4^+^ T cells, and tumor lesions show augmented NLRP3 expression in CTCL. **(A)** HTB-176 cells stimulated with PMA/ionomycin and recombinant IL-4. Representative immunofluorescence (IF) of NLRP3 (AF-488) and IL-4 (AF-594). Quantification of **(B)** NLRP3 nuclear expression and **(C)** total expression of IL-4 in the microphotographs of the different treatments (n = 3). **(D)** Representative IF for the coexpression of TOX (AF-594) and NLRP3 (AF-647) in CTCL plaques and tumors. Quantification of the total expression of **(E)** NLRP3 in the microphotographs of the plaques (n = 39) and tumors (n = 8). **(F)** Representative IF of NLRP3 (AF-488) and TOX (AF-594) expression in CD4^+^ T cells isolated from the skin of CTCL tumors (n = 3). **(G)** Representative IF for NLRP3 (AF-488) and IL-4 (AF-594) expression in plaques and tumors. **(H)** Correlation between NLRP3 and IL-4 expression from **(G)** (black dot: plaque; blue dot: tumor). The dotted line indicates the dermoepidermal junction. Arrows indicate the coexpression of markers in the different staining. Error bars = SEM. *P < 0.05; **P < 0.005; ***P < 0.001; ****P < 0.0001. Scale bars= 20 nm.

IL-4 inhibits inflammasome assembly in monocytes, thus changing NLRP3 cellular distribution (19). Therefore, IL-4 could further promote NLRP3 nuclear localization in malignant CD4^+^ T cells. Indeed, the addition of recombinant IL-4 increased NLRP3 nuclear localization and IL-4 expression (**Figures 5A–C**). Both of these characteristics were greatly boosted with the combination treatment of PMA/ionomycin and IL-4 (**Figures 5A–C**). These results suggest a positive feedback loop where IL-4 amplifies its production by favoring NLRP3 nuclear localization.

Then, we evaluated whether the positive feedback of IL-4 mediated by NLRP3 could have an impact in the late stages of the disease. We observed a higher density of TOX^+^ cells with a higher nuclear localization of NLRP3 in late-stage lesions than in early-stage lesions (**Figures 5D, E**). Likewise, nuclear localization of NLRP3 was observed in TOX^+^ CD4^+^ T cells isolated from tumor lesions (**Figure 5F**). Elevated expression of NLRP3 in cells with high expression of IL-4 was observed in the tumor stage (**Figure 5G**). Importantly, we observed a positive correlation between the expression of NLRP3 and IL-4 (**Figure 5H**). These results support the ability of IL-4 to inhibit NLRP3 inflammasome assembly, thereby promoting nuclear localization of this receptor in CTCL lesions and possibly contributing to disease progression since tumor lesions show augmented NLRP3 expression in TOX^+^ T cells.

### IL-4 Increases CTCL Malignant Features Associated With Disease Progression

**Figure 6:**
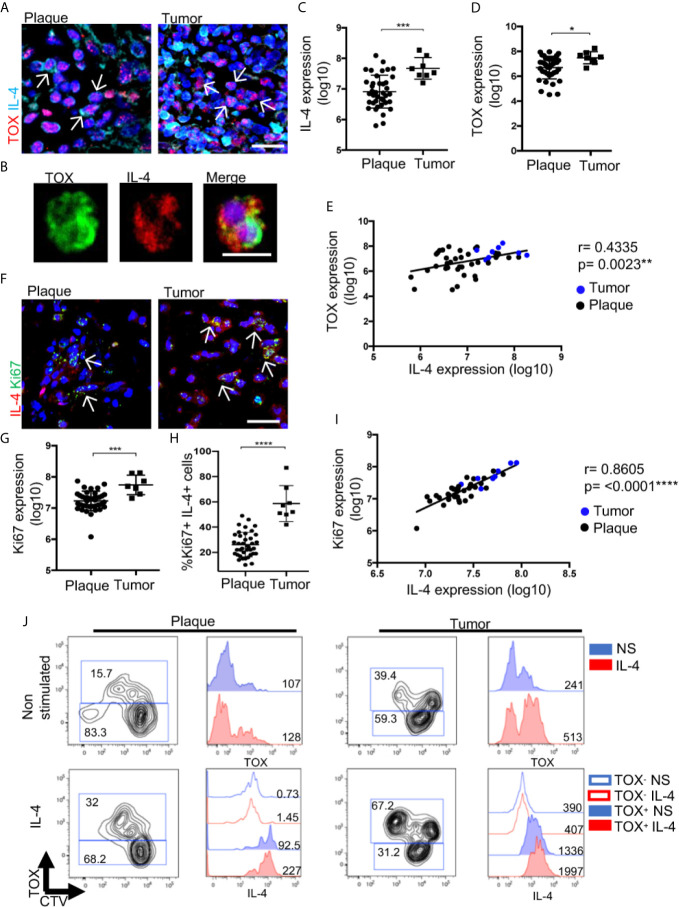
IL-4 increases CTCL features associated with disease progression. **(A)** Representative immunofluorescence (IF) of TOX (AF-594) and IL-4 (AF-647) expression in CTCL plaques and tumors. **(B)** Representative microphotographs of CD4^+^ T cells isolated from tumors showing TOX (AF-488) and IL-4 (AF-594) expression (n = 3). Quantification of the total expression of **(C)** IL-4 and **(D)** TOX in the microphotographs of plaques (n = 39) and tumors (n = 8). **(E)** Correlation between IL-4 and TOX expression quantified from **(A)** (black dot: plaque; blue dot: tumor). **(F)** Representative IF of Ki67 (AF-488) and IL-4 (AF-594) expression in CTCL plaques and tumors. Quantification of the total expression of **(G)** Ki67 and **(H)** percentage of Ki67^+^ IL-4^+^ cells in the microphotographs of CTCL plaques (n = 39) and tumors (n = 8). **(I)** Correlation between IL-4 expression and Ki67 expression quantified from **(F)** (black dot: plaque; blue dot: tumor). **(J)** Flow cytometric analysis of CTCL plaques and tumor cells treated with IL-4. Left images show the proliferating CellTrace Violet mark (CTV) and percentage of TOX^+^ cells. Right images are representative histograms of nonstimulated cells (blue, NS) or IL-4-treated cells (red, IL-4) showing the expression of TOX and IL-4 from gated CD4^+^ TOX^+^ and CD4^+^ TOX^-^ cells. Numbers represent MFI. Arrows of IF images indicate coexpression of markers in the different staining. Error bars = SEM. *P < 0.05; ***P < 0.001; ****P < 0.0001. Scale bars= 20 nm.

To evaluate the effect of IL-4 during CTCL progression from plaques to tumors, we measured the expression of IL-4 in TOX^+^ cells by comparing tumor and plaque stages (**Figure 6A**). High expression of both markers was confirmed in isolated CD4^+^ T cells from the lesion of a patient in the tumor stage (**Figure 6B**). Overall, we observed a significant increase in IL-4 and TOX expression in the tumor stage (**Figures 6C, D**), with a positive correlation between the levels of TOX and IL-4 expression (**Figure 6E**). We also observed a significant increase in the expression of IL-13 and a decrease in IFNγ in the tumor stage compared with the early stage (**Supplementary Figure 11**). Remarkably, we observed an increase in the expression of Ki67^+^ cells as well as in the percentage of TOX^+^ Ki67^+^ cells at the tumor stage compared with the plaque stage (**Figures 6F–H**), and a strong positive correlation was also observed between Ki67 and IL-4 expression (**Figure 6I**). Consistent with the pathogenic feedback loop for IL-4, cultured cells derived from a plaque or a tumor lesion significantly increased the percentage of TOX^+^ CD4^+^ cells in the presence of recombinant IL-4 (**Figure 6J**). Strikingly, even in a tumor lesion where the percentage of TOX^+^ cells was high at the basal level (40%), culturing with IL-4 still increased the expression of TOX and the percentage of TOX^+^ cells (67.2%). To recapitulate, the addition of IL-4 to cultured cells increased the production of IL-4 by TOX^+^ cells (**Figure 6J**). In addition, culturing the HTB-176 cell line in the presence of IL-4 increased the expression of TOX and Ki67 (**Supplementary Figure 12A**). These findings reinforce the notion of a positive feedback loop, which was also observed in the HTB-176 cell line, in which the blockade of IL-4 decreased both TOX expression and proliferation after PHA activation (**Supplementary Figure 12B–D**). These results strongly suggest that IL-4 increases the malignant potential of CTCL cells, thus indicating that this cytokine is central in the progression of this disease.

**Figure 7:**
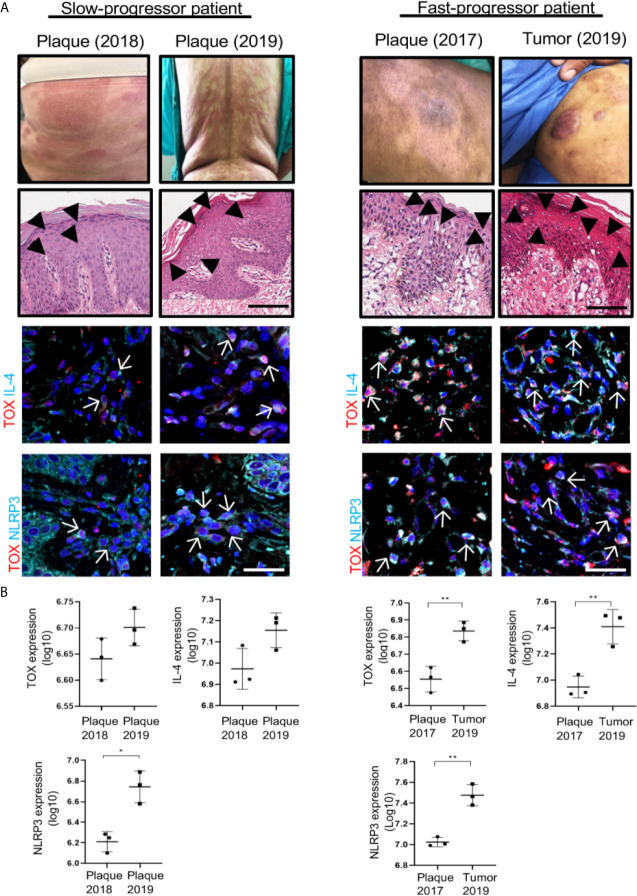
Molecules implicated in the mechanism of IL-4 regulation mediated by NLRP3 are associated with CTCL progression. **(A)** Clinical photographs of the lesions from representative slow (left) and fast (right) progressor CTCL patients. Hematoxylin-eosin (HE) staining in a slow and fast progressor CTCL patient. Representative immunofluorescence images of the expression of TOX (AF-594) and IL-4 (AF-647) (mid panel) and TOX (AF-594) and NLRP3 (AF-647) (bottom panel) in biopsies from representative slow progressor and fast progressor CTCL patients. Quantification of the total expression of **(B)** TOX, IL-4 and NLRP3 in the microphotographs of the representative slow progressor and fast progressor patient of CTCL from **(A)**. Black arrows indicate Pautrier microabscesses, and white arrows indicate coexpression of markers in the different staining. Error bars = SEM. *P < 0.05; **P < 0.01. Scale bar, 20 nm.

**Figure 8:**
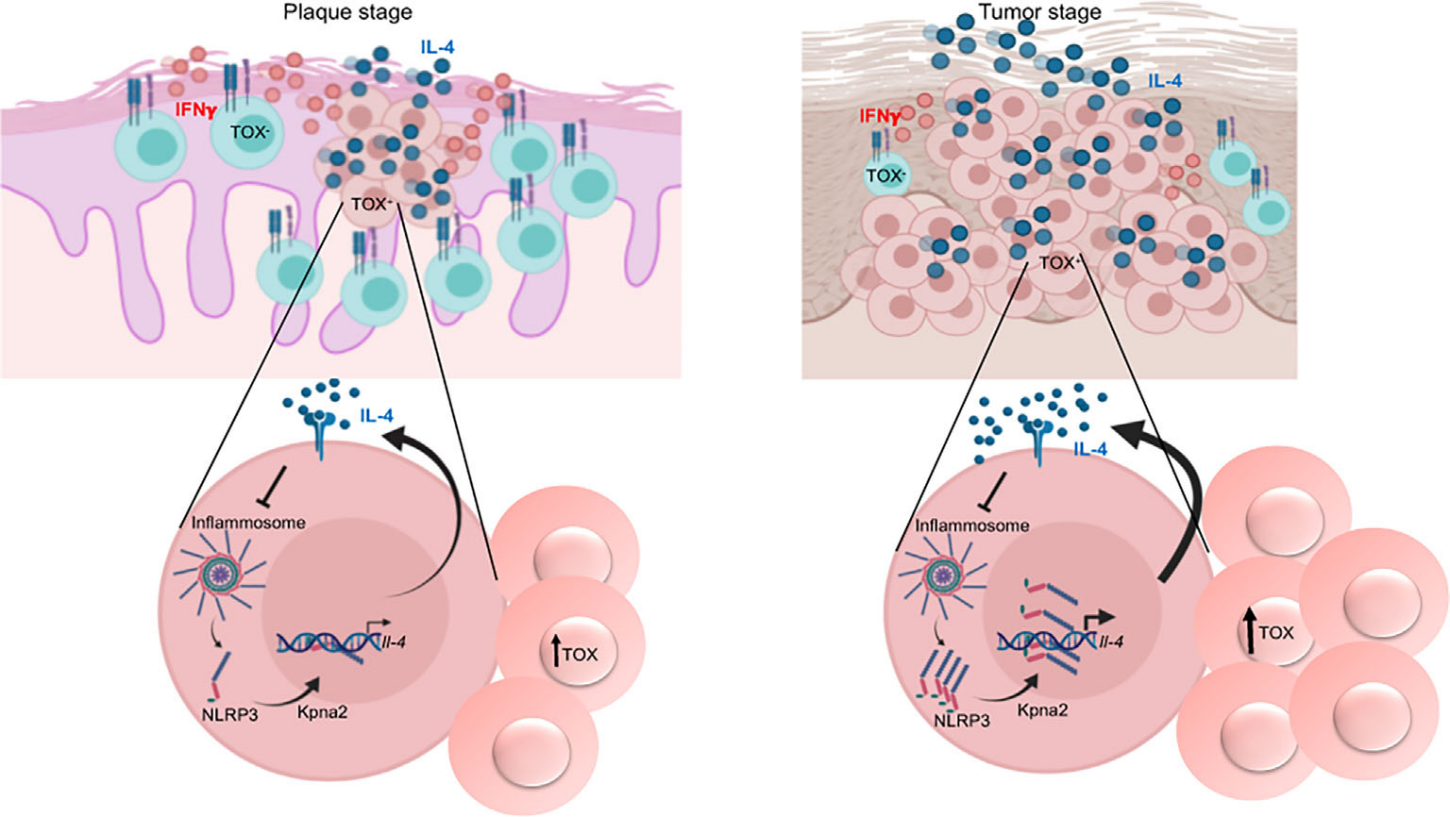
Model of the mechanism of regulation of IL-4 expression in CTCL and its role in the progression of the disease. TOX^+^ CD4^+^ T cells that express IL-4 are present beginning in the early stage of the disease. Unassembled NLRP3 translocates to the nucleus and binds to the *il-4* promoter, thus regulating IL-4 expression. Elevated levels of IL-4 then accelerate disease progression because a) NLRP3 nuclear localization is further triggered through inhibition of inflammasome assembly and b) TOX expression and TOX^+^ cell proliferation are promoted. This mechanism is amplified in the late stages of the disease, thus promoting the dominance of a Th2 milieu. Created with BioRender.com.

To demonstrate the importance of TOX, IL-4 and NLRP3 relative to the progression of the disease, we used two sequential specimens taken from two patients who presented with different speeds of disease evolution. One patient manifested as slow progressive disease (plaque to plaque), while the other manifested as fast progressive disease (plaque to tumor) (**Figure 7**). The first case was a 61-year-old female with a 9-year dermatosis that started with erythematous patches located on the forearms that spread to the trunk and extremities. In the last year, the disease evolved from small- to medium-sized plaques to large plaques that were not responsive to treatment with pegylated IFNα and methotrexate. In the first biopsy of this patient, we observed low expression of TOX, IL-4 and NLRP3, which increased in a second biopsy. In addition, we observed an increase in IL-13 and reduced expression of IFNγ (**Supplementary Figure 13**). The second case was a 59-year-old male at 10 years since disease onset. In this patient, the lesions started as hypopigmented patches with follicular accentuation. During the last 2 years, the disease evolved to erythematous-squamous plaques and tumors and was resistant to treatment with pegylated IFNα and methotrexate. The expression of TOX, NLRP3 and IL4 was significantly higher in the second biopsy, thus correlating with a progressive lesion (**Figure 7**). IL-13 expression was significantly increased in the second specimen, and IFNγ expression was significantly reduced with clinical progression (**Supplementary Figure 13**). In summary, these data support the potential role of IL-4 in CTCL progression. IL-4 seems responsible for a Th1 to Th2 cytokine switch through inhibition of the classical inflammasome function of NLRP3, and it also promotes the nuclear translocation of NLRP3 and further activation of IL-4 expression. Remarkably, this mechanism seems independent of a GATA-3-induced classical Th1 to Th2 switch. Altogether, this mechanism seems to favor expansion of potentially malignant cells, thereby increasing TOX expression and TOX^+^ cell proliferation in a dominant Th2 cytokine milieu. In this scenario, the presence of IL-4^+^ TOX^+^ CD4^+^ T cells with nuclear localization of NLRP3 appear to be a good indicator of the course of the disease (**Figure 8**).

## Discussion

In the present work, we showed that TOX^+^ CD4^+^ T cells already constitutively express IL-4 from the early stages of CTCL. Our results strongly suggest that NLRP3 is a key regulator of IL-4 in CD4^+^ T cells from CTCL lesions. We observed that IL-4 promotes a positive feedback loop by i) promoting NLRP3 nuclear localization, ii) boosting TOX expression and TOX^+^ cell proliferation, which upregulates IL-4, and iii) favoring a dominance of Th2 responses in advanced stages of the disease (tumor) (**Figure 8**). We propose that this mechanism is not markedly active at early disease stages but eventually takes control, thus igniting the progression of the disease.

Whether cytokines are secreted by reactive or malignant CD4^+^ T cells in early-stage CTCL is difficult to determine. Several reports have indicated the potential of TOX expression to identify malignant CTCL cells (7, 26, 27), although reports have also indicated that TOX is not a specific marker of malignant clones (28). We observed high IL-4 expression in TOX^+^ cells, and these cells were observed in areas of Pautrier microabscesses, which is the anatomic site where malignant T cells are in contact with Langerhans dendritic cells that provide survival signals to malignant cells (29, 30). Importantly, the expression of TOX also correlated with T cell clonality, as supported by overrepresentation of the TCRVβ22 clone in some CTCL patients. Although our results support that TOX is mainly expressed in malignant T cells in CTCL, we also observed high TOX expression in AD (31). However, the percentage of IL-4^+^ TOX^+^ cells in AD was significantly lower than that in CTCL, and the expression of TOX in AD was not restricted to CD4+ T cells. Therefore, we cannot formally rule out that some reactive cells also express TOX in the early disease stage. Consistent with our data, the findings reported for Sézary patients (14) also support the distinction of reactive (IFNγ^+^) from potentially malignant (TOX^+^ IL-4^+^) CD4^+^ T cells in CTCL patients. Furthermore, our results support the idea that the Th2 dominance observed in the tumor stage could be a consequence of the expansion of the malignant TOX^+^ IL-4^+^ T cells already present in early-stage lesions.

Compared with previous reports (32), we did not observe significant differences in the levels of GATA-3 in CD4^+^ T cells from CTCL lesions compared with CS, but its expression was significantly lower in CTCL lesions than in AD lesions. In addition, GATA-3 did not appear to be involved in the upregulation of IL-4 production in CTCL progressing lesions, thus indicating an alternative mechanism responsible for the Th2 profile. In murine Th2 cells, the free NLRP3 receptor, which separated from the other inflammasome components, can be translocated into the nucleus, bind to IRF4 and act as a TF regulator of Th2 locus expression (17). Consistent with these findings, we found that NLRP3 localizes to the nucleus of CTCL TOX^+^ cells.

We also demonstrated that NLRP3 binds to two different sites within the *il-4* promoter in the CTCL cell line HTB-176. We also found an active site for IRF4 very close to the binding site that appeared to present the highest efficiency. We described these sites for the first time in the human *il-4* promoter. However, whether NLRP3 binds to IRF4 to promote TF activity in TOX^+^ CD4^+^ T cells of CTCL in a manner similar to human M2 macrophages and mouse Th2 cells (17, 18) or whether NLRP3 activates the *il-4* promoter by itself remain to be experimentally demonstrated. Nonetheless, concomitant silencing of NLRP3 reduced IL-4 expression. These results strongly indicate that the expression of IL-4 in T cells from CTCL lesions is importantly mediated by nuclear NLRP3, particularly in more advanced disease.

We also showed high perinuclear and nuclear expression of karyopherin α2 cells in CTCL lesions. Similar to a report in mice, the regulation of IL-4 expression in CD4^+^ T cells from CTCL was dependent upon the unassembled form of NLRP3. This finding was confirmed with the use of an NLRP3 assembly inhibitor, which increased NLRP3 nuclear localization, favored the binding of NLRP3 to the IL-4 promoter and correlated with an increase in IL-4 expression. Although the signals that triggered NLRP3 translocation to the nucleus are unknown, the fact that we observed high perinuclear and nuclear expression of NRLP3 in CTCL but not in AD strongly suggests that the nuclear form of NRLP3 is associated with the transformation of CD4+ T cells. However, it is still very important to further define the signals that trigger the nuclear localization of NLRP3, identify how the manipulation of those stimuli affects cell proliferation, and determine the expression of Th2 cytokines and markers of cell transformation, in which other diseases related to a Th2 profile could be active. Adding another layer of complexity, we showed that IL-4 inhibits cytoplasmic NLRP3 inflammasome assembly, which is similar to observations in human monocytes (19). Therefore, we propose a positive feedback loop in which IL-4 favors its own production via NLRP3 in CD4^+^ T cells, thereby further enriching a Th2 environment in CTCL lesions.

Compared with our findings, two recent reports showed that NLRP3 suppresses innate and adaptive Th2 immune responses in a model of helminth infection (33, 34). This finding suggests that NLRP3 displays different functions depending on different factors, such as the insult, microenvironment, cell or tissue type, and cell activation or differentiation status.

Remarkably, we observed increased nuclear NLRP3 expression in tumor lesions and during disease progression, which was also strongly correlated with IL-4 expression. These results strongly suggest that nuclear NLRP3 plays a key role in expanding the production of IL-4 during disease progression. This Th2 response dominates the later stages of the disease and could inhibit the Th1 response, which is similar to what has been reported in Sézary patients (14). We found that IL-4 increases malignant features, such as proliferation and TOX expression. Accordingly, it was recently reported that IL-13 and IL-4 increase the proliferation of CD4^+^ T cells isolated from the blood of Sézary patients (12). Importantly, we observed an increase in the expression of TOX in the presence of IL-4 and a positive correlation between IL-4 and TOX, which suggested the coregulated expression of these molecules during disease progression. Altogether, these observations are critical in the context of cancer progression because TOX has been reported to have an oncogenic role by promoting cellular proliferation, metastasis and resistance to cellular death by apoptosis (6, 27). These findings provide important insights into the molecular mechanisms underlying the pathogenesis of CTCL, which may also be active in other T cell lymphomas. Consequently, the expression of IL-4 and the nuclear localization of NLRP3 in combination with TOX positivity could have clinical applications as potential prognostic markers, and this mechanism of IL-4/NLRP3/TOX enhancement may provide an effective target in the design of new therapeutic strategies to stop CTCL progression.

## Data Availability Statement

The original contributions presented in the study are included in the article/**Supplementary Material**. Further inquiries can be directed to the corresponding author.

## Ethics Statement

The studies involving human participants were reviewed and approved by Comisión Nacional de Investigación Científica, Instituto Mexicano del Seguro Social, Mexico. Written informed consent to participate in this study was provided by the participants’ legal guardian/next of kin. Written informed consent was obtained from the individual(s) for the publication of any potentially identifiable images or data included in this article.

## Author Contributions

Conceptualization, LB and EH-M. Formal analysis, EH-M. Funding acquisition, LB. Methodology and main experiments, EH-M. Supporting experiments, MA-H, BH-R, GV-A, AP-G, and PM-C. Project administration, LB. Resources, LB. Diagnoses and recruited all CTCL patients, MD-G, FJ-S, and AL-L. Confocal microscopy support, VP-K and GP-L. Supervision, PL-L and LB. Writing original draft, EH-M and LB. EM-P discussed and revised the results and thoroughly revised the manuscript. Writing-review and editing, LB, MA-H, and LL-P. All authors contributed to the article and approved the submitted version.

## Funding

This study was funded by Fondo SEP-CONACYT CB-2017-2018- A1-S-16141 (to LB). EH-M received a scholarship (616824) from the *Consejo Nacional de Ciencia y Tecnología (CONACyT)* in México.

## Conflict of Interest

The authors declare that the research was conducted in the absence of any commercial or financial relationships that could be construed as a potential conflict of interest.

## Abbreviations

CTCL, Cutaneous T cell lymphoma; NLRP3, NLR Family Pyrin Domain Containing 3; Th, T helper, CS, Control skin.
