# Supplementary material for: NLRP3 Regulates IL-4 Expression in TOX+ CD4+ T Cells of Cutaneous T Cell Lymphoma to Potentially Promote Disease Progression
Source: Front Immunol. 2021 Jun 16;12:668369. doi: 10.3389/fimmu.2021.668369 (PMC8244903; doi:10.3389/fimmu.2021.668369)
Supplement: Supplementary file 1 [file DataSheet_1.pdf]

Supplementary Material

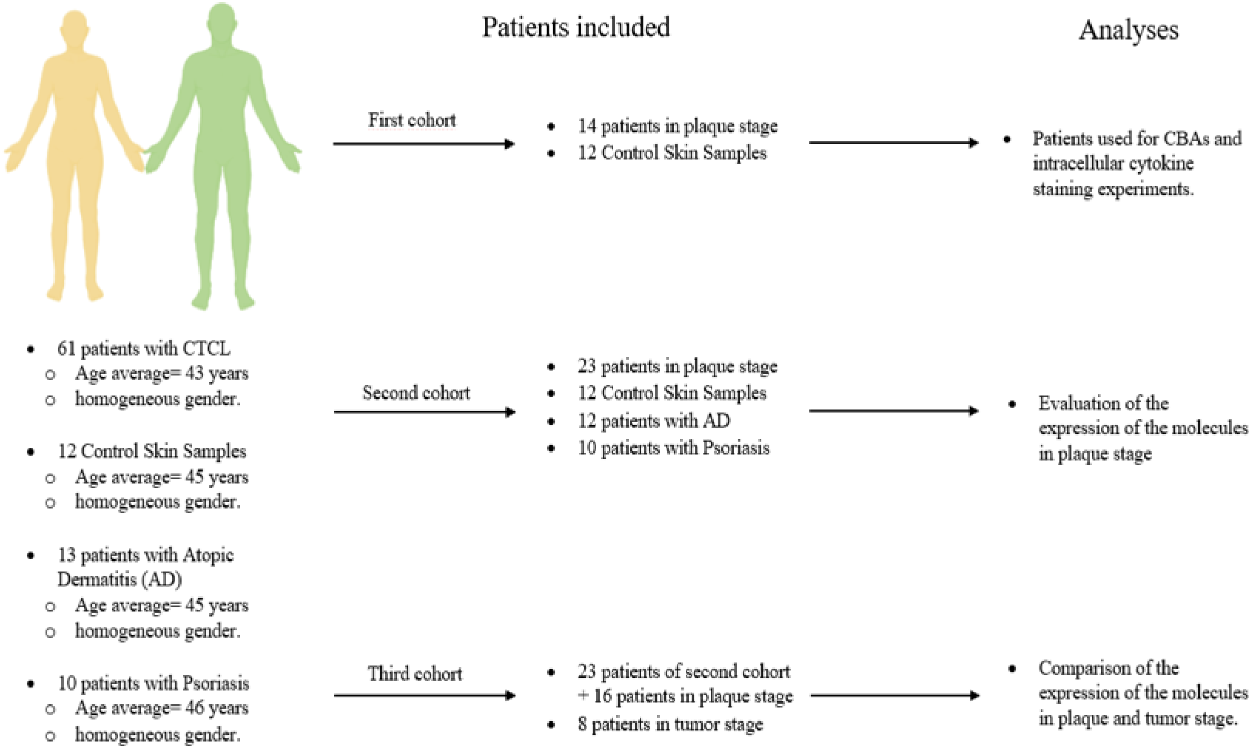

Supplementary Figure 1. Study design for CTCL patients and controls.

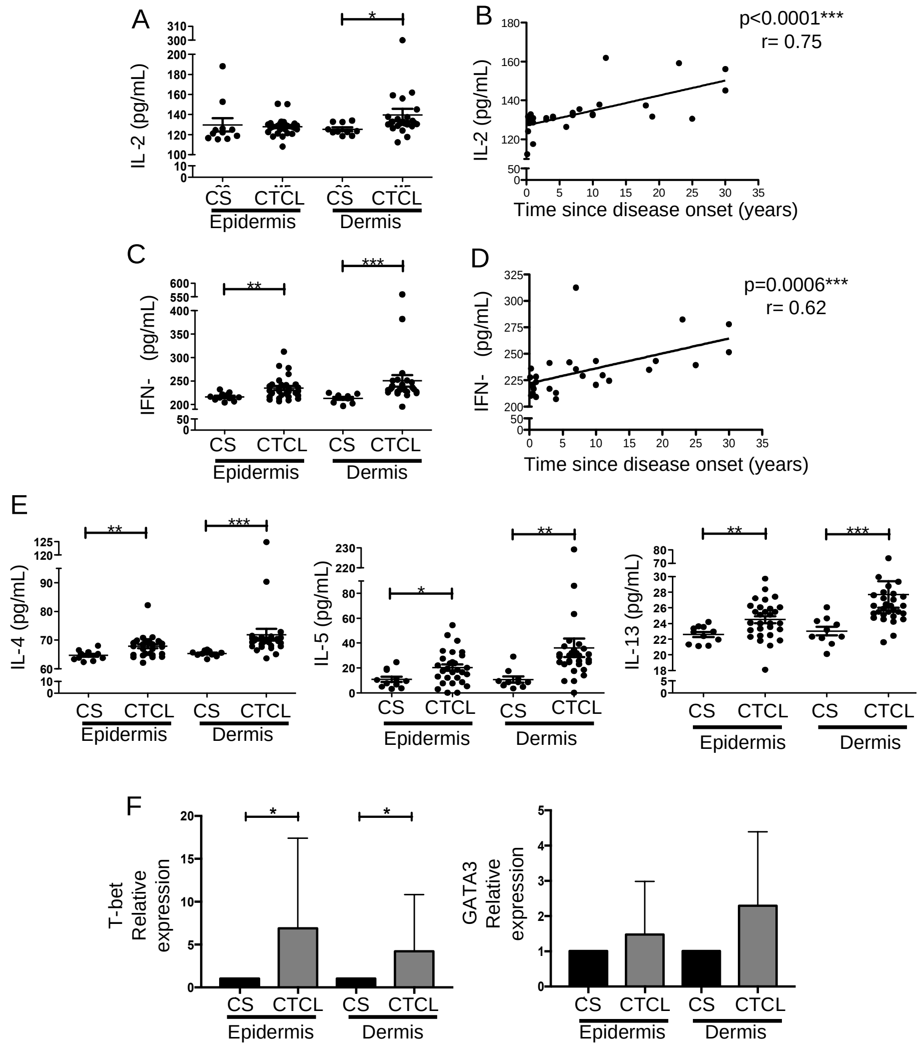

Supplementary Figure 2. Skin lesions of early stage Cutaneous T Cell Lymphoma patients contain a mixed Th1/Th2 cytokine profile.

Cytokines were quantified using Flow Cytomix Multiplex in the supernatants of dermis and epidermis cultures of skin from CTCL plaques (n=23) and CS (n=10). (**A**) Quantification of IL-2. (**B**) Correlation between time since disease onset (years) and levels of IL-2. (**C**) Quantification of IFN-γ. (**D**) Correlation between time since disease onset (years) and IFN-γ expression. (**E**) Quantification of IL-4, IL-5 e IL-13. (**F**) Expression of T-bet and GATA-3 in $CD3^+$ $CD4^+$ T cells in CS (black bars) and the skin of CTCL patients (grey bars) measured by flow cytometry. Bars show mean fluorescence intensity (MFI) in $CD3^+$ $CD4^+$ T cells from CTCL plaques relative to the average in CS. The mean of data was obtained, and statistical significance was calculated using Mann-Whitney's t-test. Error bars = SEM. *P<0.05; **P<0.005; ***P< 0.001.

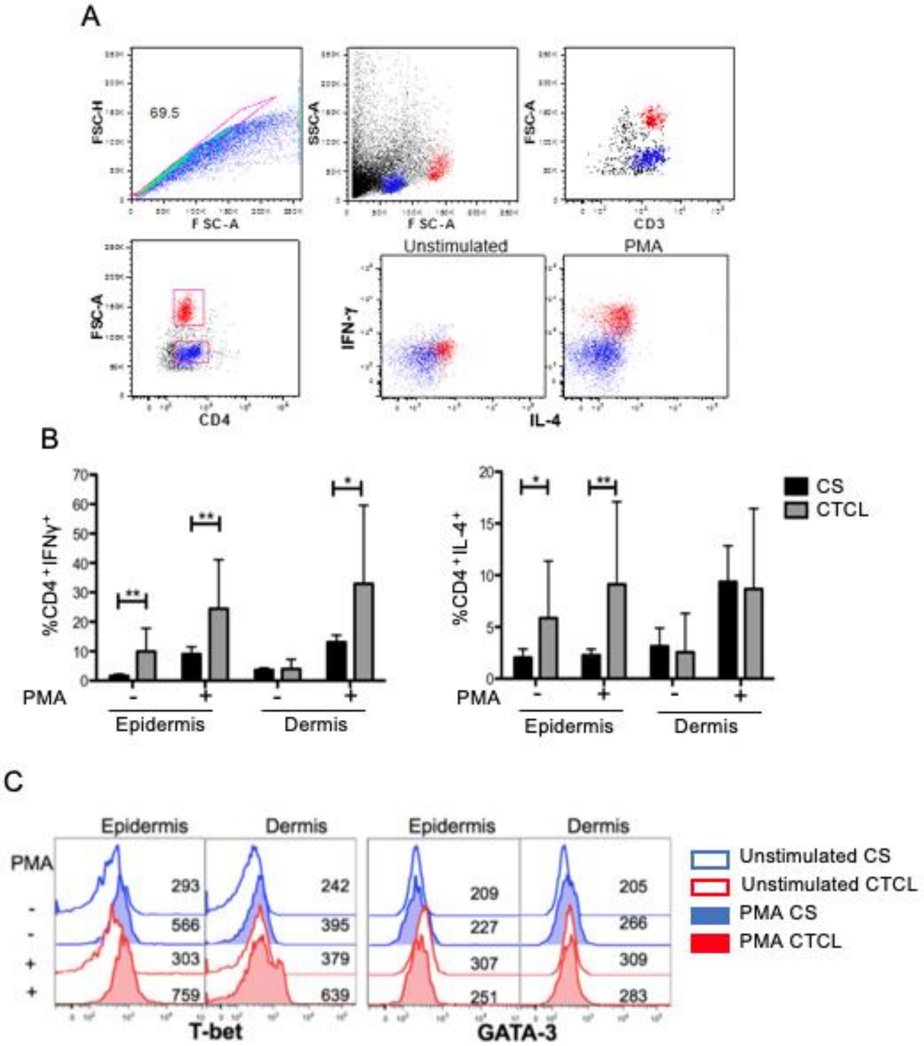

Supplementary Figure 3. Skin lesions of early-stage Cutaneous T Cell Lymphoma patients contain large atypical CD4$^+$ IL-4$^+$ T cell in the epidermis.

Intracellular cytokines staining in CD3$^+$ CD4$^+$ cells by flow cytometry. (**A**) IFN-γ and IL-4 expression in CD4$^+$ cells from CTCL plaque (n=14). (**B**) Percentage of IFNγ$^+$ CD4$^+$ and IL-4$^+$ CD4$^+$ cells in the dermis and epidermis cultures of CTCL plaques (n=14) and CS (n=6). (**C**) Histograms of T-bet and GATA-3 in skin T cells. Total cells were recovered after 72 h of culture and left unstimulated (empty histograms) or stimulated with PMA/Iono for 6 hours (filled histograms), in CTCL plaques (red) and CS (blue) (n=5). The mean of data was obtained, and statistical significance was calculated using Mann-Whitney's t-test. Error bars = SEM. *P<0.05; **P< 0.01.

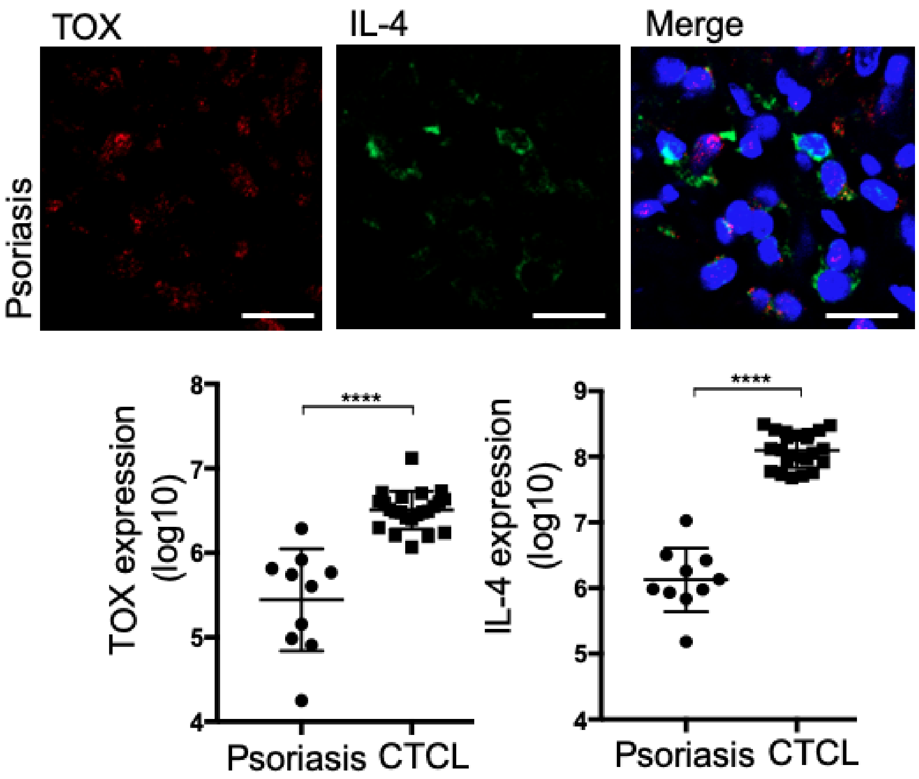

Supplementary Figure 4. TOX and IL4 are not overexpressed in psoriasis.

Immunofluorescence of TOX (AF-594) and IL-4 (AF-488) in skin lesions derived from psoriasis patients, a benign inflammatory disease. Quantification of the total expression of TOX and IL-4 in CTCL plaques (n=23) and psoriasis (n=10). Arrows indicate coexpression of markers in the different staining. Error bars = SEM. ****P<0.0001.

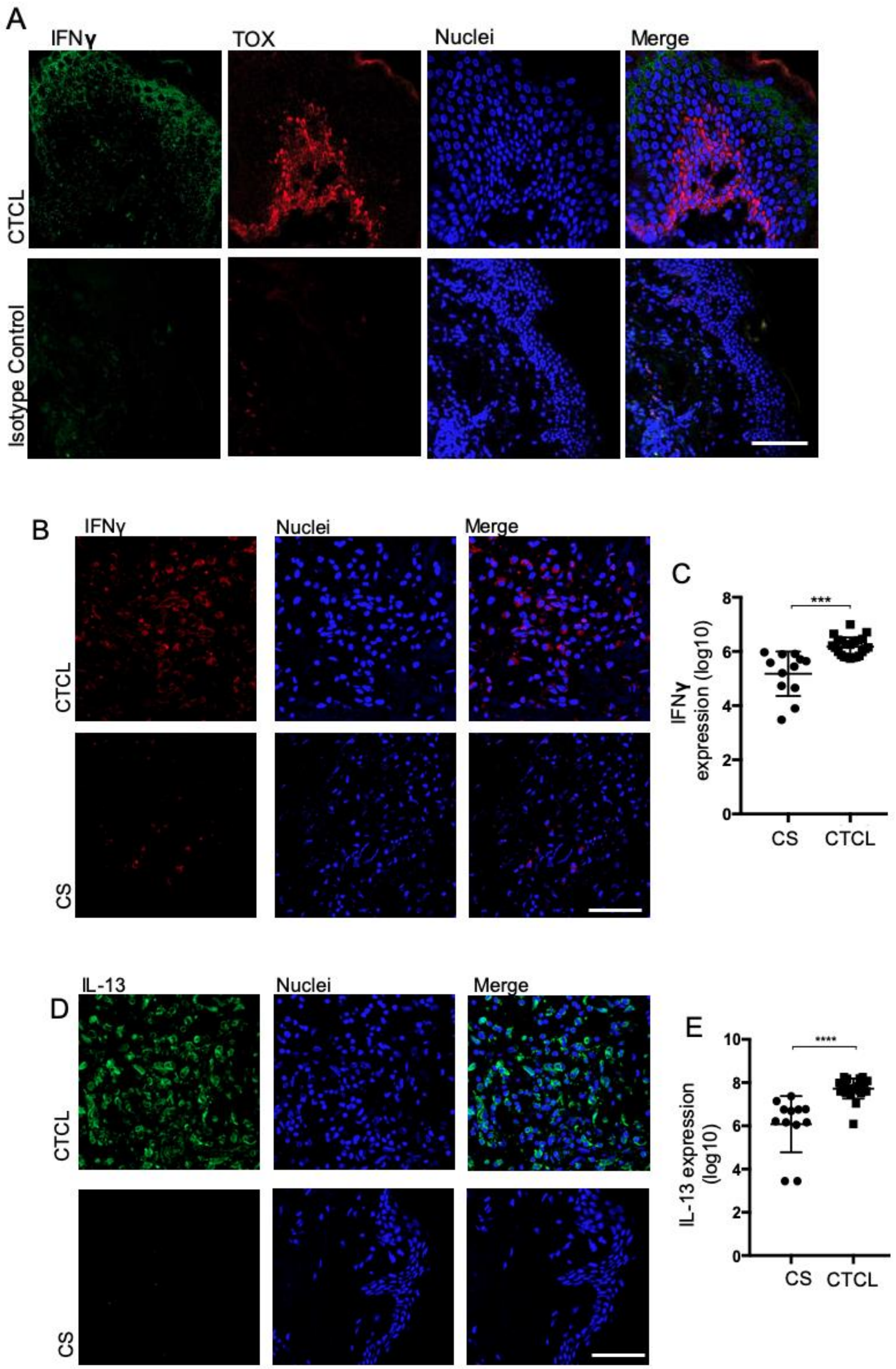
A
IFNγ
TOX
Nuclei
Merge
CTCL
Isotype Control
B
IFNγ
Nuclei
Merge
CTCL
CS
C
***
IFNγ expression (log10)
8
6
4
2
0
CS
CTCL
D
IL-13
Nuclei
Merge
CTCL
CS
E
****
IL-13 expression (log10)
10
8
6
4
2
0
CS
CTCL

Supplementary Figure 5. IFNγ is expressed by TOX⁻ T cells in CTCL plaques.

(**A**) Representative immunofluorescence microphotographs of the TOX (AF-594) and IFNγ (AF-488) expression in CTCL plaques. (**B**) IFNγ (AF-594) and (**D)** IL-13 (AF-488) expression. Quantification of the total expression of (**C**) IFNγ and (**E**) IL-13 in the microphotographs of CTCL plaques (n=23) and CS (n=12). Error bars = SEM. ***P<0.001; ****P<0.0001. Scale bar= 20 nm.

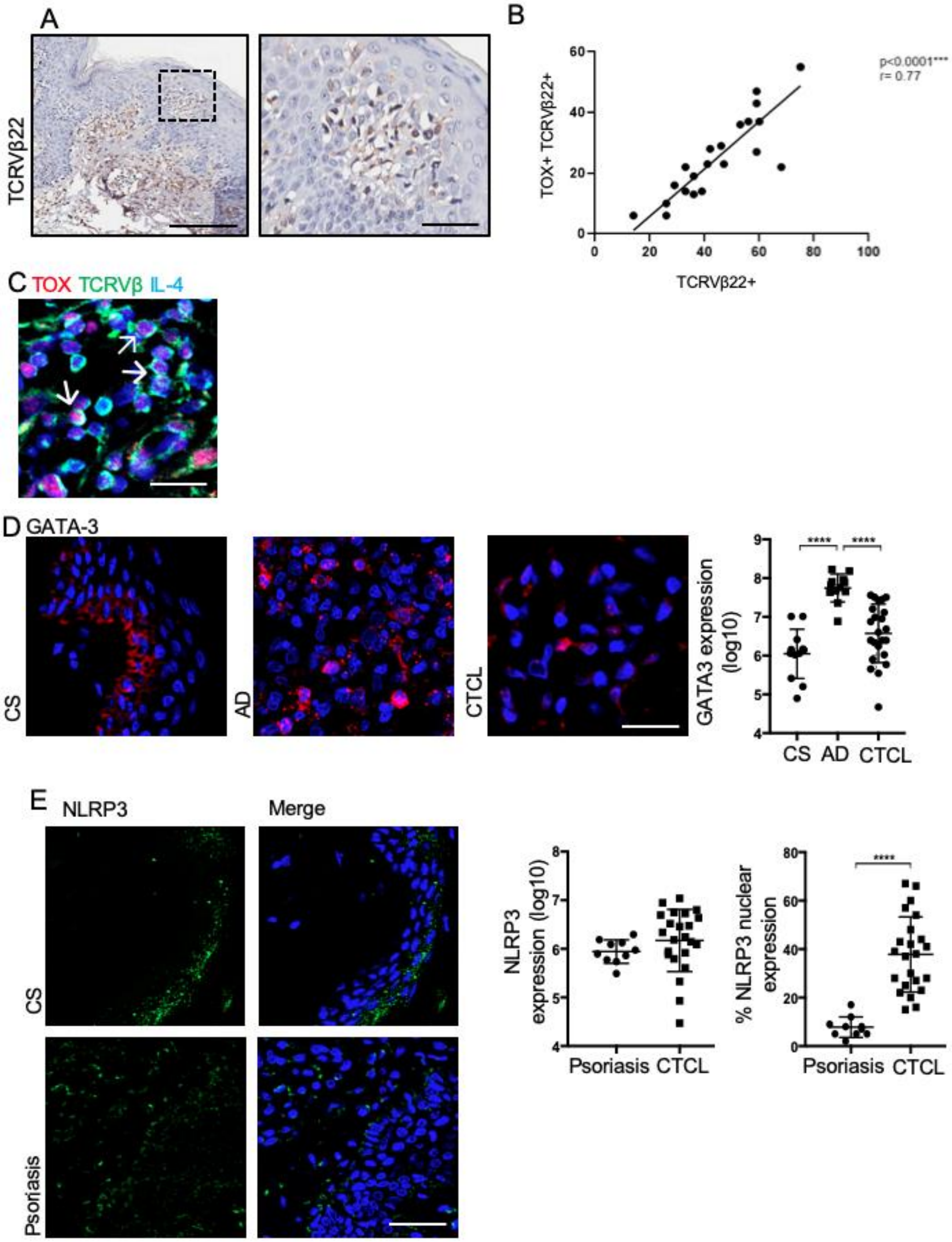

Supplementary Figure 6. IL-4 is mainly produced by TOX^+ $CD4^+$ T cells associated with clonality in skin lesions of CTCL patients in early stages.

(A) Immunohistochemistry (IHC) of the expression of TCRVβ22 in Pautrier microabscesses. (**B**) Correlation between the percentage of TCRVβ22 and the percentage of cells that express both TOX and TCRVβ22 (n=23) from (B). (**C)** Representative immunofluorescence (IF) images of TOX (AF-594), TCRVβ22 (AF-488) and IL-4 (AF-647) in plaques of CTCL. (**D**) Representative IF depicting GATA3 (AF-594) expression in CTCL plaques and CS. Quantification of the total expression of GATA3 in CTCL plaques (n= 23), AD (n=12) and CS (n= 12). (**E**) Representative IF of NLRP3 (AF-488) in psoriasis and CS. Quantification of the total expression of NLRP3 and the percentage of cells with nuclear NLRP3 in psoriasis (n=10) and CTCL plaques (n=23). Error bars = SEM. ***P<0.001, ****P<0.0001. Scale bar= 20 nm.

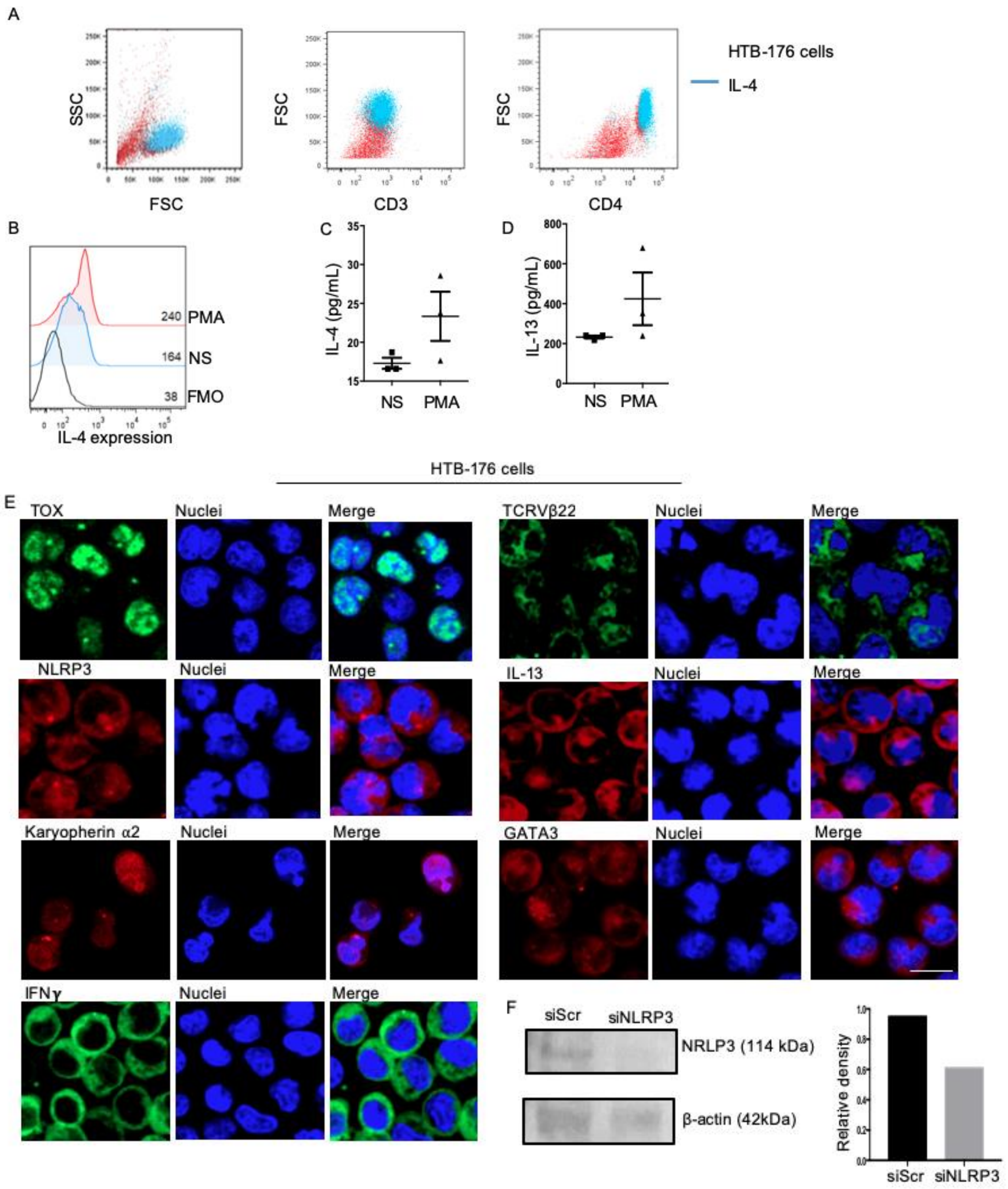

Supplementary Figure 7. HTB-176 cell line characterization.

(**A**) Representative dot plot showing IL-4 production, IL-4^{+} cells are shown in blue. (**B**) Representative histogram of IL-4 in HTB-176 cells. (**C**) Quantification of the expression of IL4 and (**D**) IL-13 in the supernatant of HTB-176 cultures (n=3) from (B). (**E**) Representative immunofluorescence microphotographs of TOX (AF-488), NLRP3 (AF-594), Karyopherin α2 (AF-594), IFNγ (AF-488), TCRVβ22 (AF-488), IL-13 (AF-594) and GATA3 (AF-594). (**F**) NLRP3 expression was evaluated by Western blot after NLRP3 silencing with specific

siRNA. Graph showing WB analysis and densitometry. Error bars = SEM. Scale bar, 20 nm. NS, non-stimulated; FMO, fluorescence minus one, siNLRP3, siRNA targeting NLRP3; siScr, siRNA scrambled control; MFI, median fluorescence intensity.

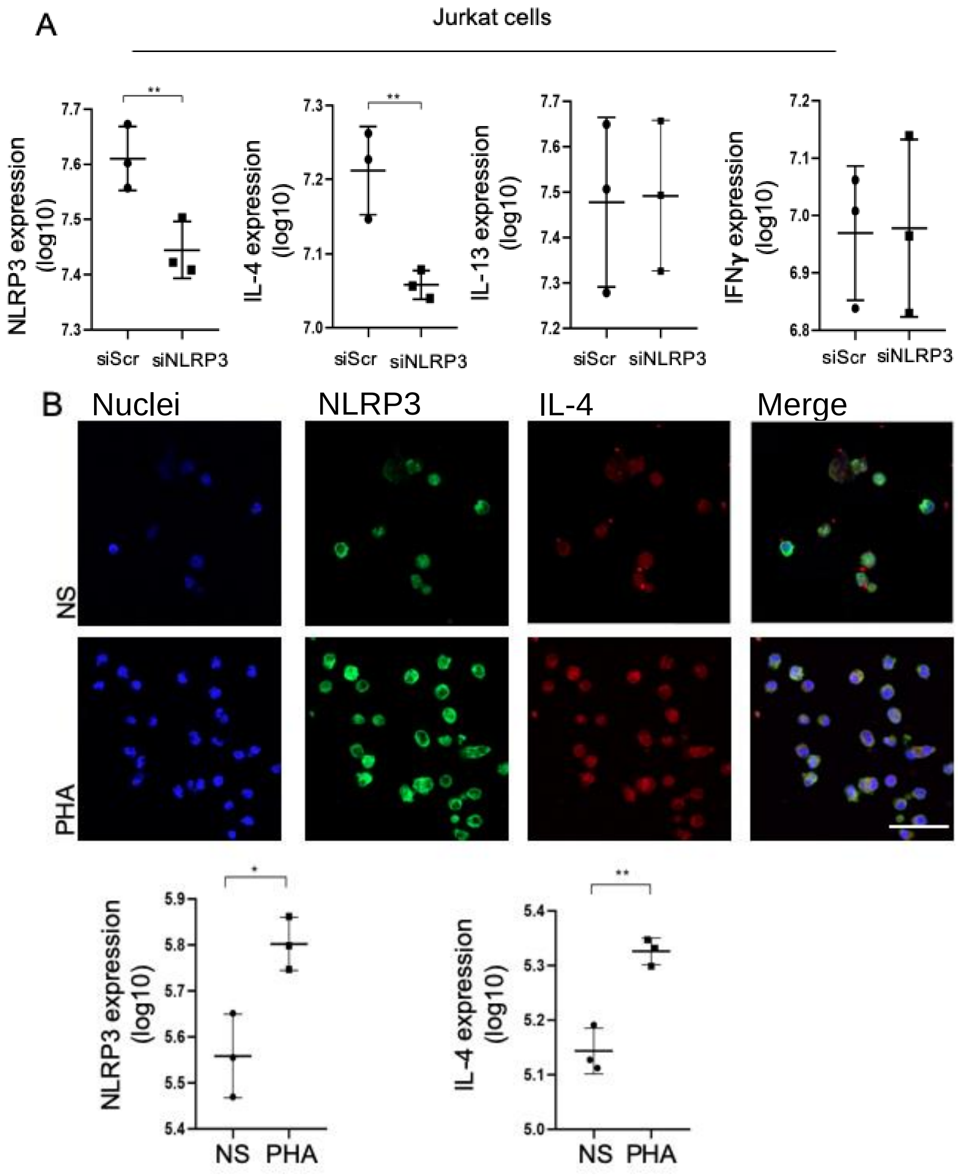

Supplementary Figure 8. Expression of NLRP3 and IL-4 are correlative in T cell lines.

(**A**) The Jurkat cell line was transfected with siNLRP3 and siScr using Lipofectamine (n=3). Quantification of the total expression of NLRP3, IL-4, IL-13 and IFNγ from immunofluorescence microphotographs. (**B**) Representative Immunofluorescence of NLRP3

(AF-488) and IL-4 (AF-594) in HTB-176 cells treated with phytohemagglutinin (PHA). Quantification of the total expression of NLRP3 and IL-4 after treatment (n=3). Error bars = SEM. *P<0.05; **P<0.01. Scale bar= 20 nm.

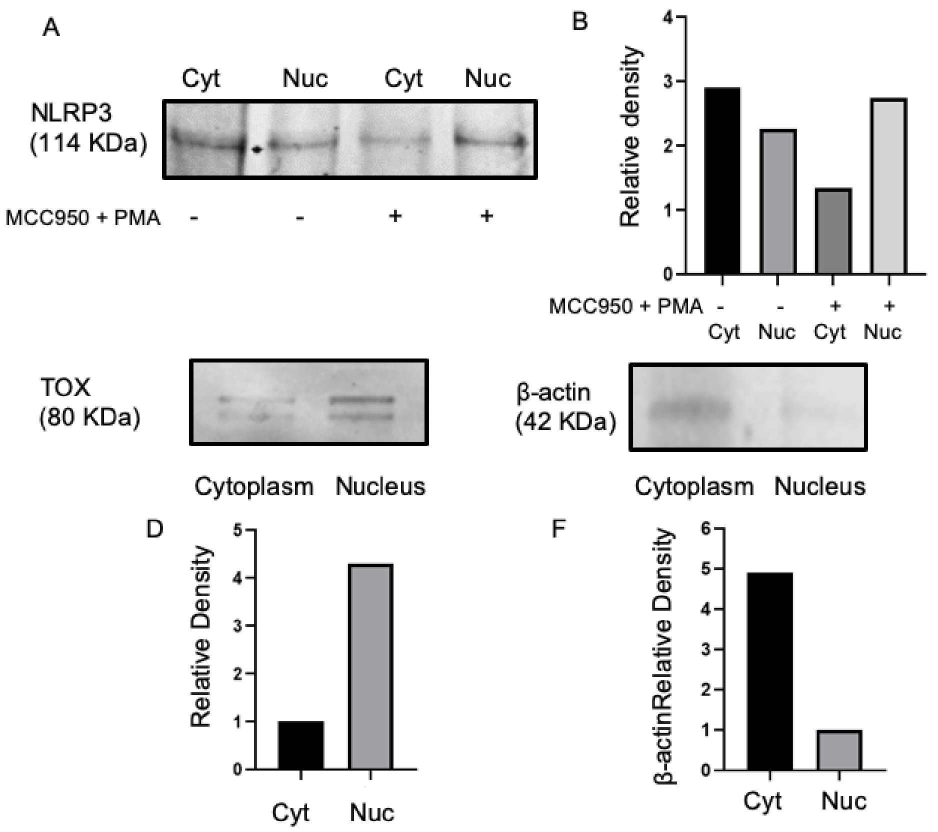

Supplementary Figure 9. Nuclear localization of NLRP3 increases by inhibiting the assembly of the inflammasome. (A) Evaluation of the expression of NLRP3 by Western blot (WB) in the cytoplasmic and nuclear fractions of cell extracts of the HTB-176 cell line treated or not with the inhibitor MCC950 + PMA. **(B)** Quantification of the relative density of NLRP3 cytoplasmic and nuclear fractions in each condition. **(C)** Evaluation of the expression of TOX by WB in extracts corresponding to the cytoplasm and nuclear fractions. **(D)** Quantification of the relative density of TOX in each subcellular fraction. (**E)** Evaluation of the expression of β-actin by WB in extracts corresponding to the cytoplasm and nuclear fractions. **(F)** Quantification of the relative density of β-actin in each subcellular fraction.

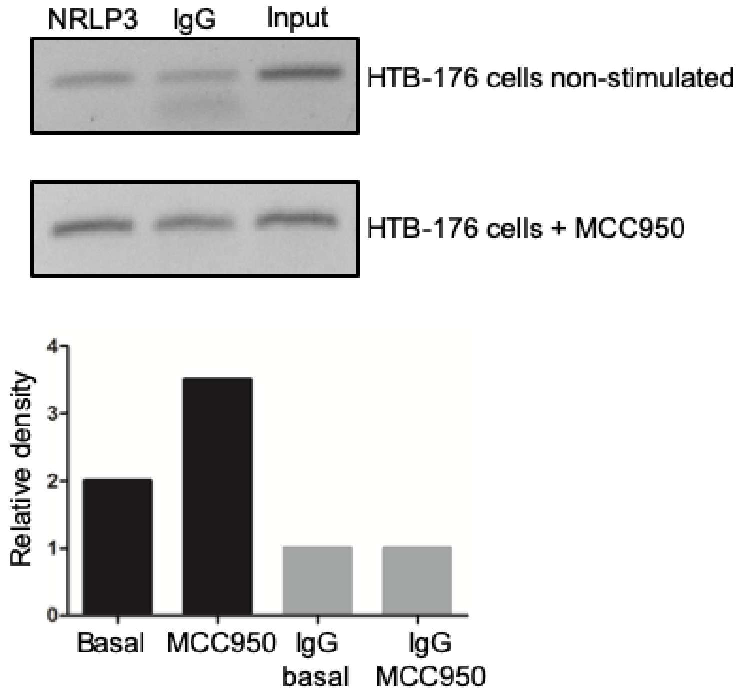

Supplementary Figure 10. Inflammasome inhibition increases NLRP3 binding to the *il-4* promoter in HTB-176 cells.

ChIP assays were performed using an NRLP3 specific antibody. The binding of NRLP3 to the NRLP3-1 site in the *il-4* promoter region of HBT-176 cells was estimated in basal or inflammasome inhibiting conditions.

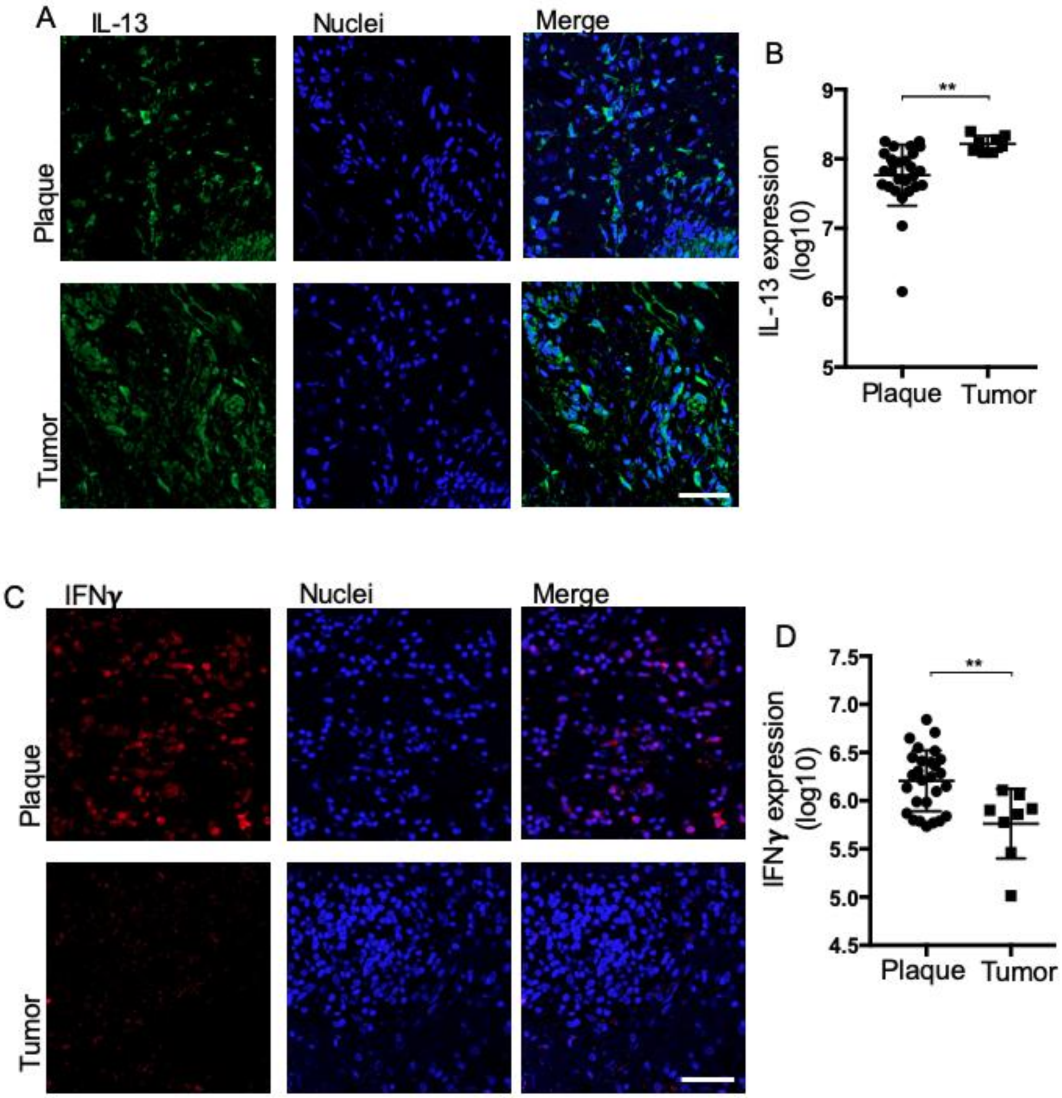

Supplementary Figure 11. In tumor stage of CTCL there is an increase of IL-13 and IFNγ is decreased.

Representative microphotographs of the IL-13 (AF-488) expression in CTCL plaque and tumor stage lesions. Quantification of the total expression of (**B**) IL-13 in the microphotographs of CTCL plaques (n=27) and tumors (n=8). (**C**) Representative microphotographs of the IFNγ (AF-594) expression in CTCL plaque and tumor stage lesions. Quantification of the total expression of (**D**) IFNγ in the microphotographs of CTCL plaques (n=27) and tumors (n=8). Error bars = SEM. *P<0.05; **P<0.01. Scale bar= 20 nm.

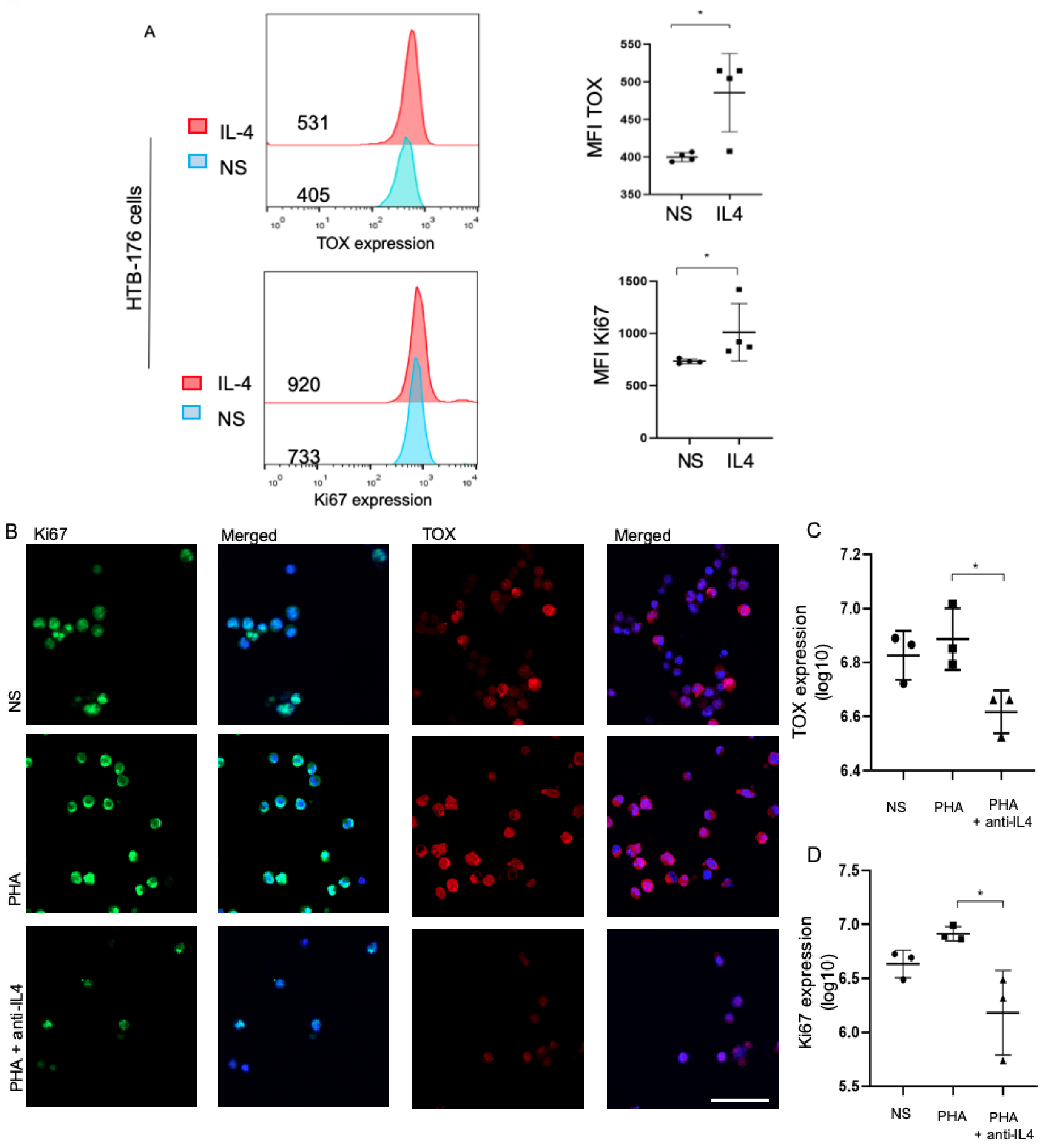

Supplementary Figure 12. IL-4 promotes TOX expression and proliferation.

(**A**) Flow cytometric analysis of the cells treated with IL-4. Histogram and quantification of the MFI of TOX and Ki67 expression (n=4). (**B**) HTB-176 cells stimulated with PHA and treated with anti-IL-4. Microphotographs depicting the expression of Ki67 (AF-488) and TOX (AF-594). Quantification of the total expression of **(C)** TOX and (**D**) Ki67 in the

microphotographs after the different treatments (n=3). Error bars = SEM. *P<0.05. Scale bar= 20 nm. NS, Non-stimulated; PHA, phytohemagglutinin.

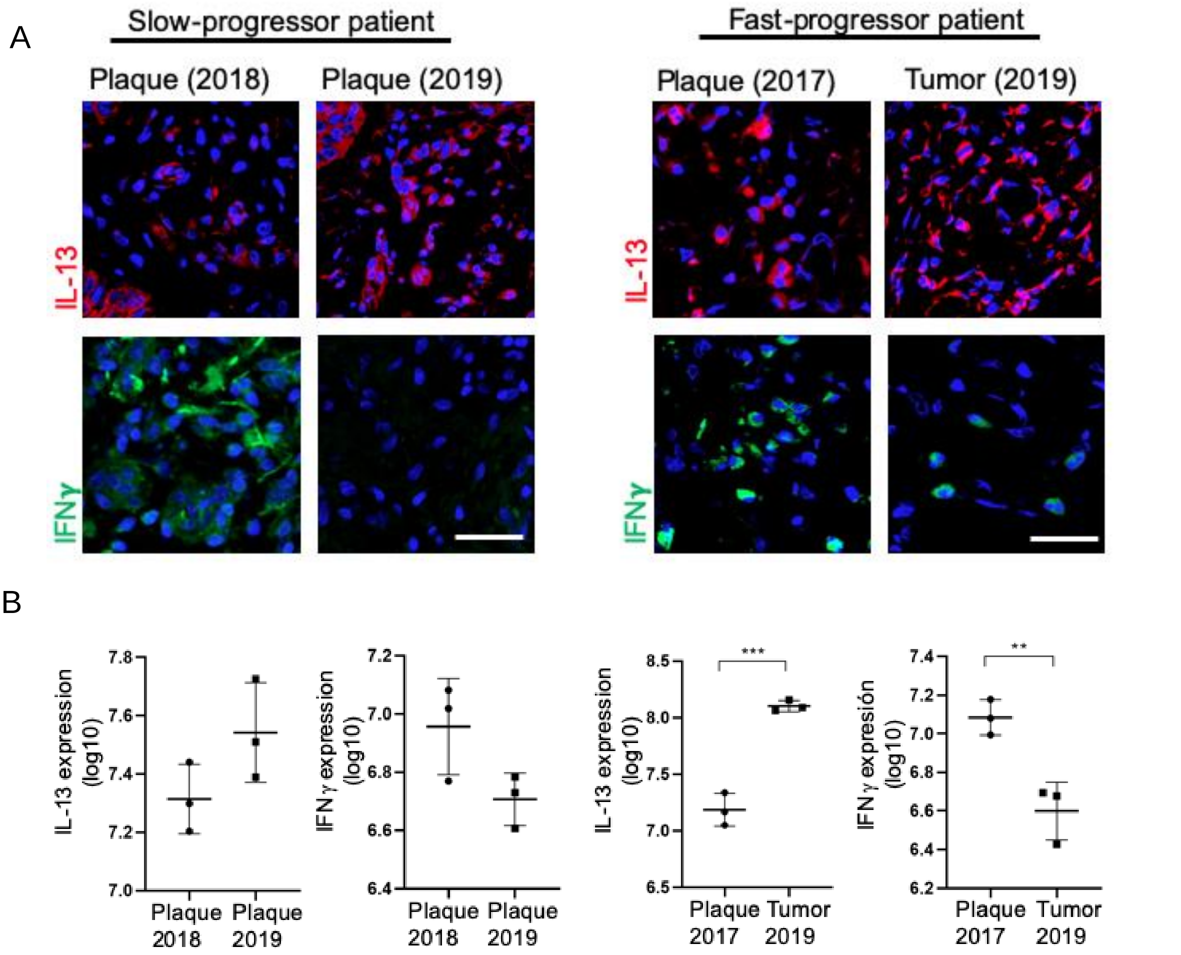

Supplementary Figure 13. Increased IL-13 and decreased IFNγ associated with CTCL progression.

(A) Representative Immunofluorescence images of the expression of IL-13 (AF-594) and IFNγ (AF-488) in biopsies from representative slow progressor and fast progressor CTCL patients. Quantification of the total expression of **(B)** IL-13 and IFNγ in the microphotographs of representative slow progressor and fast progressor CTCL patients. Error bars = SEM. **P<0.01, ***P<0.001. Scale bar= 20 nm.

Supplementary video 1. Reconstruction of Z-stack image from a CTCL plaque stage biopsy. Reconstruction of Z-stack images of the expression of NLRP3 (AF-488) and Karyopherin α2 (AF-594) in CTCL plaque biopsy.

Supplementary video 2. Reconstruction of Z-stack image from an Atopic Dermatitis biopsy. Reconstruction of Z-stack images of the expression of NLRP3 (AF-488) and Karyopherin α2 (AF-594) in AD biopsy.

Supplementary video 3. Reconstruction of Z-stack image from HTB-176 cells. Reconstruction of Z-stacks image of the expression of NLRP3 (AF-488) in the HTB-176 cells nonstimulated with MCC950 inhibitor.

Supplementary video 4. Reconstruction of Z-stack image from HTB-176 cells. Reconstruction of Z-stacks image of the expression of NLRP3 (AF-488) in the HTB-176 cells after treatment with MCC950 inhibitor plus PMA.

Supplementary Table 1. Demographic and clinical data of CTCL patients with tissue paraffin-embedded

	Personal Data		Stage	Time of disease evolution (years)	Affected Body Surface Area (%)	TNM	WHO-EORTC
	Gender	Age					
1	Masculine	47	Plaque	8	39.5	IB	MF
2	Feminine	63	Plaque	2.5	78	IB	MF
3	Masculine	57	Plaque	7	40	IB	MF
4	Feminine	9	Plaque	NA	37	IB	MF
5	Feminine	28	Plaque	1	19.75	IB	MF
6	Masculine	41	Plaque	5	50	IB	MF
7	Masculine	20	Plaque	8	24.5	IB	MF
8	Feminine	29	Plaque	10	39	IB	MF
9	Masculine	37	Plaque	0.25	59	IB	Folliculotropic MF
10	Feminine	23	Plaque	5	26	IB	MF
11	Feminine	72	Plaque	10	54.5	IB	MF
12	Feminine	9	Plaque	9	21	IB	MF
13	Feminine	29	Plaque	7	35.5	IB	MF
14	Masculine	41	Plaque	1	68	IB	MF

15	Masculine	25	Plaque	2	79	IB	MF
16	Feminine	64	Plaque	30	76	IB	MF
17	Masculine	40	Plaque	8	73	IB	MF
18	Masculine	59	Plaque	19	33	IB	MF
19	Feminine	65	Plaque	60	25	IB	MF
20	Feminine	58	Plaque	0.25	25.75	IB	MF
21	Masculine	43	Plaque	0.67	39	IB	Folliculotropic MF
22	Feminine	61	Plaque	7	80	IIA	MF
23	Feminine	60	Plaque	11	70	IIA	MF
24	Feminine	14	Plaque	0.13	59	IB	MF
25	Masculine	25	Plaque	10	75	IB	MF
26	Masculine	19	Plaque	1	20	IB	MF
27	Masculine	37	Plaque	NA	3.5	IA	MF
28	Masculine	35	Plaque	2	13.5	IB	MF
29	Masculine	49	Plaque	20	45	IB	MF
30	Feminine	38	Plaque	12	28	IB	MF
31	Feminine	18	Plaque	15	60.25	IB	MF
32	Masculine	40	Plaque	6	40	IB	MF
33	Feminine	48	Plaque	20	81	IIA	MF

34	Masculine	34	Plaque	10	50	IB	MF
35	Masculine	30	Plaque	2	22.5	IB	MF
36	Masculine	39	Plaque	5	60	IIA	MF
37	Masculine	57	Plaque	2	1	IA	MF
38	Feminine	59	Plaque	5	72	IB	MF
39	Feminine	39	Plaque	5	60	IIA	MF
40	Masculine	57	Plaque and tumor	39	40	IIB	Folliculotropic MF
41	Masculine	48	Tumor	1	5	IIB	MF
42	Feminine	65	Tumor	3	100	IVB	SS
43	Feminine	73	Tumor	4	80	III	MF
44	Masculine	42	Tumor	2	20	IIB	MF
45	Feminine	60	Tumor	11	70	IIB	MF
46	Masculine	39	Tumor	7	60	IIB	MF
47	Masculine	41	Tumor	5	50	IIB	Folliculotropic MF

NA: Not Available

Supplementary Table 2. Demographic and clinical data of CTCL patients without tissue paraffin-embedded

	Personal Data						
	Gender	Age	Stage	Time of disease evolution (years)	Affected Body Surface Area (%)	TNM	WHO-EORTC
1	Feminine	18	Plaque	9	68	IB	MF
2	Masculine	64	Plaque	10	38	IB	MF
3	Masculine	38	Plaque	0.5	64	IB	MF
4	Feminine	9	Plaque	4	20	IB	MF
5	Feminine	59	Plaque	19	33	IB	MF
6	Masculine	63	Plaque	0.167	85.5	IIA	MF
7	Masculine	78	Plaque	9	80	IIA	MF
8	Feminine	41	Plaque	4	93	IIA	MF
9	Masculine	43	Plaque	0.667	39	IB	MF
10	Masculine	38	Plaque	0.5	64	IB	MF
11	Masculine	12	Plaque	4	33	IB	MF
12	Feminine	32	Plaque	12	55	IB	MF
13	Feminine	54	Plaque	26	40	IB	MF
14	Masculine	69	Plaque	40	29	IB	MF

Patients used for CBA and intracellular cytokine staining experiments for whom we do not have available tissue paraffin-embedded

Supplementary Table 3. Antibodies used for flow cytometry and immunofluorescence.

Antigen	Manufacturer	Clone	Catalog Number	RRID	Isotype	Fluorochrome/Use
CD3	Biolegend	OKT3	317307	AB_571912	Mouse IgG2a kappa	Phycoerythrin
CD4	BD Bioscience	RPA-T4	560158	AB_1645478	Mouse IgG1 kappa	APCH7
IL-4	Biolegend	MP4-25D2	500822	AB_961404	Mouse IgG1 kappa	PerCP-Cy5.5
T-bet	Biolegend	4B10	644805	AB_1595593	Mouse IgG1 kappa	PerCP-Cy5.5
GATA3	BD Bioscience	L50-823	560163	AB_1645302	IgG1	Alexa Fluor 488
IFNγ	BD Bioscience	B27	557643	AB_396760	Mouse IgG1 kappa	PECy7
TOX	eBioscience	TXRX10	50-6502-82	AB_2574265	Rat IgG2a kappa	eFluor 660
Ki67	Biolegend	Ki67	350522	AB_2563863	Mouse IgG1 kappa	Brilliant Violet 605
IL-4	Santa Cruz Biotechnologies, TX, USA	C-19	sc-1260	AB_2128970	Goat polyclonal	IF
IL-4	RyD Systen, MI, USA	#3007	MAB304	AB_2889404	Mouse monoclonal	Neutralization
IL-13	Abcam, Cambridge, UK	Polyclonal	ab106732	AB_10867235	Rabbit polyclonal	IF

TOX	Thermo Scientific, MA, USA	Polyclonal	PA5-30328	AB_2547802	Rabbit polyclonal	IF, WB, IHC
TCRVβ22	Beckman Coulter, CA, USA	IMMU 546 human	IM1484	AB_131022	Mouse monoclonal	IF, FC, IHC
IFNγ	Biolegend, CA, USA	MD-1	507501	AB_2122340	Mouse monoclonal	IF
NLRP3	RyD System, MI, USA	#768319	MAB7578	AB_2889405	Rat monoclonal	IF, WB
Karyopherin α2	BD Bioscience, CA, USA	C-2	610485	AB_397855	Mouse monoclonal	IF
GATA-3	Abcam, Cambridge, UK	Polyclonal	ab106625	AB_10887935	Rabbit polyclonal	IF
IRF4	Biolegend, CA, USA	IRF4.3E4	646411	AB_2728477	Rat IgG1 kappa	ChIP
Rat IgG (H+L)	Jackson Immunoresearch, PA, USA	Polyclonal	712-585-153	AB_2340689	Donkey polyclonal	IF, AF 594
Rabbit IgG (H+L)	Jackson Immunoresearch, PA, USA	Polyclonal	711-545-152	AB_2313584	Donkey polyclonal	IF, AF 488
Rabbit IgG (H+L)	Jackson Immunoresearch, PA, USA	Polyclonal	711-585-152	AB_2340621	Donkey polyclonal	IF, AF 594
Goat IgG (H+L)	Jackson Immunoresearch, PA, USA	Polyclonal	705-065-147	AB_2340397	Donkey polyclonal	IF, AF 647
Mouse IgG (H+L)	Jackson Immunoresearch, PA, USA	Polyclonal	715-605-151	AB_2340863	Donkey polyclonal	IF, AF 647

Rat IgG (H+L)	Thermo Scientific, MA, USA	Polyclonal	A-11006	AB_2534074	Goat polyclonal	IF, AF 488
Rabbit IgG Bond Polymer Refine Detection	Leica Biosystems	Polyclonal	DS9800	AB_2891238	Goat polyclonal	IHC
Mouse/Rabbit ImmunoDetector Link & AP Label	Bio Science	Polyclonal	BSB-0351	AB_2891237	Goat polyclonal	IHC
Beta-Actin	GeneTex	Polyclonal	GTX109639	AB_1949572	Rabbit polyclonal IgG	WB
IRDye 800CW Goat anti-Rat	LI-COR Biosciences	Polyclonal	926-32219,	AB_1850025	Goat polyclonal IgG	WB
IRDye 800CW Goat anti-Rabbit	LI-COR Biosciences	Polyclonal	926-32211	AB_621843	Goat polyclonal IgG	WB

Supplementary Table 4. Primers used for PCR

Gene	Forward primer	Reverse Primer
NLRP3 (site 1)	ATACCACATGATCTCACGCATA	CACCATGTCGTACAATGGCT
NLRP3 (site 3)	CAGGTGCCTGTAGTCCCAGCT	AGTGCAGTGGCGTGATCTTAGTTC
IRF4	GTGACAGAGCAAGATTCCATCT	TGGACACAATAAGGTGCTCATT
IgF2	CAGGCTCCCCCAAAATCTA	GGGAACATAGAGAAAGAGG
